# Shp-2 is critical for ERK and metabolic engagement downstream of IL-15 receptor in NK cells

**DOI:** 10.1038/s41467-019-09431-3

**Published:** 2019-03-29

**Authors:** Charlène Niogret, S. M. Shahjahan Miah, Giorgia Rota, Nicolas P. Fonta, Haiping Wang, Werner Held, Walter Birchmeier, Veronica Sexl, Wentian Yang, Eric Vivier, Ping-Chih Ho, Laurent Brossay, Greta Guarda

**Affiliations:** 1 Department of Biochemistry, University of Lausanne, 1066 Epalinges, Switzerland; 2 Department of Molecular Microbiology and Immunology and Graduate Program in Pathobiology, Division of Biology and Medicine, Brown University Alpert Medical School, Providence, RI 02912 USA; 3 Università della Svizzera italiana (USI), Faculty of Biomedical Sciences, Institute for Research in Biomedicine, 6500 Bellinzona, Switzerland; 4 Department of Oncology UNIL CHUV, University of Lausanne, 1066 Epalinges, Switzerland; 5 Department of Fundamental Oncology, University of Lausanne, 1066 Epalinges, Switzerland; 6 Max-Delbrueck-Center for Molecular Medicine (MDC) in the Helmholtz Society, 13125 Berlin, Germany; 7 Department for Biomedical Sciences, Institute of Pharmacology and Toxicology, University of Veterinary Medicine, 1210 Vienna, Austria; 8 Department of Orthopaedics, Rhode Island Hospital and Brown University Alpert Medical School, 1 Hoppin Street, Providence, RI 02903 USA; 9 Centre d’Immunologie de Marseille-Luminy, Aix Marseille Université, Inserm, CNRS, Avenue de Luminy, 13288 Marseille, France; 10 Service d’Immunologie, Hôpital de la Timone, Assistance Publique-Hôpitaux de Marseille, 13385 Marseille, France; 11 Innate Pharma Research Labs., Innate Pharma, 117 Avenue de Luminy, 13276 Marseille, France

## Abstract

The phosphatase Shp-2 was implicated in NK cell development and functions due to its interaction with NK inhibitory receptors, but its exact role in NK cells is still unclear. Here we show, using mice conditionally deficient for Shp-2 in the NK lineage, that NK cell development and responsiveness are largely unaffected. Instead, we find that Shp-2 serves mainly to enforce NK cell responses to activation by IL-15 and IL-2. Shp-2*-*deficient NK cells have reduced proliferation and survival when treated with high dose IL-15 or IL-2. Mechanistically, Shp-2 deficiency hampers acute IL-15 stimulation-induced raise in glycolytic and respiration rates, and causes a dramatic defect in ERK activation. Moreover, inhibition of the ERK and mTOR cascades largely phenocopies the defect observed in the absence of Shp-2. Together, our data reveal a critical function of Shp-2 as a molecular nexus bridging acute IL-15 signaling with downstream metabolic burst and NK cell expansion.

## Abstract (web summary)

The phosphatase Shp-2 was implicated in NK cell education due to its reported association with inhibitory receptors, but its function in this context is unclear. Here the authors show that Shp-2 is not required for NK cell function, but is necessary for IL-15-induced metabolic burst and expansion.

## Introduction

Natural killer (NK) cells are innate cytotoxic lymphocytes poised to eliminate infected or transformed cells. NK cell effector functions are tightly regulated by the balance of activating and inhibitory signals. In healthy individuals, the balance is dominated by inhibitory signals. This is accomplished via the recognition of self-molecules, such as major histocompatibility (MHC) class I, by a variety of inhibitory receptors expressed at their cell surface^1,2^. Interestingly, inhibitory receptor engagement by MHC class I molecules, somewhat counterintuitively, is also required during NK cell development for conferring optimal NK cell function^1–4^. In support of this, NK cells from MHC class I-deficient animals are largely hyporesponsive to activating receptor-mediated stimuli^4–6^. NK cell inhibitory receptor signaling is mediated by phosphatases, including the SH2-containing inositol phosphatase-1 (SHIP-1) and the crucial SH2 domain-containing tyrosine phosphatase-1 (Shp-1)^7–11^, which is recruited to the inhibitory receptor immunoreceptor tyrosine-based inhibitory motifs (ITIM) upon engagement. Confirming the critical role of inhibitory receptor engagement in NK cell education, Shp-1-deficient NK cells are hyporesponsive.

Shp-2 (encoded by the gene *Ptpn11*) is a tyrosine phosphatase that—together with its close relative Shp-1—belongs to the family of SH2 domain-containing protein tyrosine phosphatases (PTPs)^12^. Both phosphatases bear two SH2 domains in their N-terminal part followed by a PTP domain responsible for the catalytic activity. In the inactive state, the SH2 domains bind the PTP region, thereby blocking access of substrates to the active site. This auto-inhibition is relieved upon binding of the SH2 domains to phosphotyrosine residues on targets, including ITIMs of inhibitory receptors, rendering the catalytic site accessible^12^.

As shown by the embryonic lethality of full-body deleted mice, Shp-2 plays essential developmental functions, being involved in cell growth, differentiation, proliferation, and survival^12^. In humans, SHP-2 loss-of-function results in Leopard syndrome, a severe disease marked by skeletal malformation, short stature, heart disease and mental retardation. SHP-2 gain-of-function mutations result in Noonan syndrome, which shares clinical features with Leopard syndrome^13^. Shp-2 exerts atypical effects, as it has mainly been ascribed positive roles in regulating growth factor and hormone receptor signaling. The best-characterized functions of Shp-2 are the positive regulation of the mitogen-activating protein kinases (MAPK) and—to some extent—the modulation of the phosphoinositide 3-kinase (PI3K)-Akt and mammalian target of rapamycin (mTOR) pathway downstream of various growth factor receptors in different tissues^12,14–18^. Shp-2 has therefore been proposed to dephosphorylate negative regulators of the MAPK pathway^12^. Along these lines, SHP-2 overactivation is involved in cancer development and novel inhibitors have been developed to treat these malignancies^19,20^. Last, as SHP-2 has been shown to interact with the ITIMs of NK cell inhibitory receptors^21,22^, an inhibitory role in NK cells was predicted. However, this assumption has not been experimentally confirmed.

Because Shp-2 is required for embryonic development, we generate mice conditionally deficient for Shp-2 in the NK lineage. In contrast to Shp-1 and SHIP-1 phosphatases, we find that Shp-2 is largely dispensable for NK cell education. Instead, the absence of Shp-2 is disadvantageous when NK cells are exposed to high IL-15 doses. This phenotype is linked to a markedly impaired engagement of ERK and to a compromised metabolic activation. In agreement with these findings, Shp-2-deficient Ly49H^+^ NK cells have defective proliferation during MCMV infection. Taken together, these data uncover an unanticipated role of Shp-2 in NK cells, identifying this phosphatase as an essential node in NK cell metabolism downstream of the IL-15 receptor.

## Results

### Ncr1cre Ptpn11^fl/fl^ mice exhibit increased NK cell numbers

*Ptpn11* is expressed in NK cells, as shown by measuring transcript abundance in developing bone marrow (BM) as well as splenic NK cells of intermediate CD27^+^CD11b^+^ (DP) and mature CD27^−^CD11b^+^ (CD11b SP) stage (Supplementary Fig. 1a). We thus sought to determine the contribution of Shp-2 to NK cell development and functions by generating two independent NK cell-specific knockout lines. In one case, *Ptpn11*^fl/fl^ (exons 3 and 4) mice were crossed to the *Ncr1cre*^Ki^ deleter strain (knock-in for a Cre recombinase under the control of the *Ncr1* promoter, which is active in NK cells at the immature-mature stage; hereafter referred to as *Ncr1*^Ki^ *Ptpn11*^fl/fl^)^15,23^. This led to a virtually complete Shp-2 deletion in NK cells (Supplementary Fig. 1b). In the second case, *Ptpn11*^fl/fl^ (exon 11) mice were bred with the *Ncr1cre*^Tg^ deleter strain (transgenic for Cre under the control of the *Ncr1* promoter; hereafter referred to as *Ncr1*^Tg^ *Ptpn11*^fl/fl^)^24,25^. As the efficiency of Cre-mediated deletion in this model was not complete, these mice were further crossed to a reporter strain expressing YFP upon Cre recombinase activity, which faithfully paralleled Shp-2 deletion (Supplementary Fig. 1c).

**Fig. 1:**
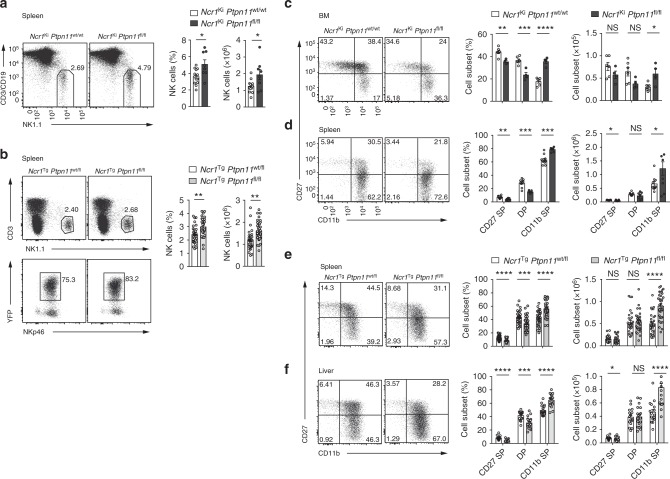
Loss of Shp-2 leads to increased mature NK cell numbers. **a**, **b** Representative flow cytometric image, frequency, and number of splenic NK cells (gated as NK1.1^+^CD3/CD19^−^) of *Ncr1*^Ki^ *Ptpn11*^fl/fl^ (dark gray) and control mice (white) are depicted (**a**) or of splenic NK cells (gated as NK1.1^+^CD3^−^YFP^+^NKp46^+^) of *Ncr1*^Tg^ *Ptpn11*^fl/fl^ (light gray) or *Ncr1*^Tg^ *Ptpn11*^wt/fl^ (white) mice (**b**) are depicted. **c**, **d** Analysis of NK cells from *Ncr1*^Ki^ *Ptpn11*^fl/fl^ and control mice. **c** Representative cytometric plot of BM NK cells (gated as CD122^+^NK1.1^+^NKp46^+^CD3/CD19^−^) stained with CD27 and CD11b; percentage and number of CD27 single-positive (CD27 SP; CD27^+^CD11b^−^), double-positive (DP; CD27^+^CD11b^+^), and CD11b single-positive (CD11b SP; CD27^−^CD11b^+^) populations are shown. **d** Representative cytometric plot of splenic NK cells (gated as NK1.1^+^ NKp46^+^CD3/CD19^−^) stained with CD27 and CD11b; percentage and number of the afore-mentioned subsets are shown. **e** As in (**d**), but for NK cells gated as NK1.1^+^CD3^−^YFP^+^NKp46^+^ from *Ncr1*^Tg^ *Ptpn11*^fl/fl^ or *Ncr1*^Tg^ *Ptpn11*^wt/fl^ mice. **f** Frequency and number of CD27 SP, DP, and CD11b SP conventional NK cells (gated as CD45^+^NK1.1^+^CD3^−^YFP^+^NKp46^+^DX5^+^CD49a^−^) from *Ncr1*^Tg^ *Ptpn11*^fl/fl^ and control mice in the liver and a representative staining of CD27 and CD11b expression. **a**, **b** Results represent the mean ± SEM of *n* = 8 (*Ncr1*^Ki^ *Ptpn11*^fl/fl^) and *n* = 13 (*Ncr1*^Ki^ *Ptpn11*^wt/wt^) (**a**), *n* = 29 (*Ncr1*^Tg^ *Ptpn11*^wt/fl^) and *n* = 35 (*Ncr1*^Tg^ *Ptpn11*^fl/fl^) (**b**) mice per genotype, are a pool of three (**a**) and nine (**b**) experiments, which are representative of at least five (**a**) and eleven (**b**) independent experiments. **c**–**f** Results represent mean ± SEM of *n* = 4 (*Ncr1*^Ki^ *Ptpn11*^fl/fl^) and *n* = 6 (*Ncr1*^Ki^ *Ptpn11*^wt/wt^) (**c**), *n* = 7 (*Ncr1*^Ki^ *Ptpn11*^fl/fl^) and *n* = 10 (*Ncr1*^Ki^ *Ptpn11*^wt/wt^) (**d**), *n* = 29 (*Ncr1*^Tg^ *Ptpn11*^wt/fl^) and *n* = 35 (*Ncr1*^Tg^ *Ptpn11*^fl/fl^) (**e**), and *n* = 16 (*Ncr1*^Tg^ *Ptpn11*^wt/fl^) and *n* = 20 (*Ncr1*^Tg^ *Ptpn11*^fl/fl^) (**f**) mice per genotype, are a pool of two (**d**), nine (**e**, **f**) experiments and representative of at least three (**c**, **d**), and eleven (**e**, **f**) independent experiments. Statistical comparisons are shown; *p ≤ 0.05, **p ≤ 0.01, ***p ≤ 0.001, ****p ≤ 0.0001, NS, non-significant; Student’s *t*-test. Source data are provided as a Source Data file

We next assessed the abundance of NK lymphocytes in the spleen of *Ncr1*^Ki^ *Ptpn11*^fl/fl^ and *Ncr1*^Tg^ *Ptpn11*^fl/fl^ mice. Interestingly, and in contrast to Shp-1 deficient animals^7^, we found that NK cell frequency and number were higher in mice conditionally deficient for Shp-2 in the NK lineage than in controls (Fig. 1a, b). We next assessed whether this phenotype was observed during their development. No significant differences were observed in percentages and numbers of CD122^+^ NK cells, as well as in their developmental stages, as measured by the sequential acquisition of NK1.1 and DX5 in the bone marrow (BM) of *Ncr1*^Ki^ *Ptpn11*^fl/fl^ mice or *Ncr1*^Ki^ *Ptpn11*^wt/wt^ controls (Supplementary Fig. 1d and e). However, maturation of Shp-2-deficient BM NK cells was slightly altered, exhibiting increased percentages and numbers of the most mature CD11b SP subset (Fig. 1c). We also analyzed the maturation status of NK lymphocytes in spleen and liver. Similar to the BM, frequency and number of CD11b SP NK cells were higher than in controls in the spleen of both Shp-2-deficient models, and in the liver (Fig. 1d, e, and f). In the liver, similarly to other organs, conventional NK cell frequency and number were increased. In contrast, ILC1 (CD49a^+^DX5^−^) frequency and number were decreased (Supplementary Fig. 1f). Importantly, mixed BM chimeras demonstrated that Shp-2 controlled the numbers of mature NK cells in a cell-intrinsic manner (Supplementary Fig. 1g). Taken together, data from two independent mouse models show that Shp-2 limits the number of the most mature NK cell subset.

### Shp-2-deficient NK cells are educated

**Fig. 2:**
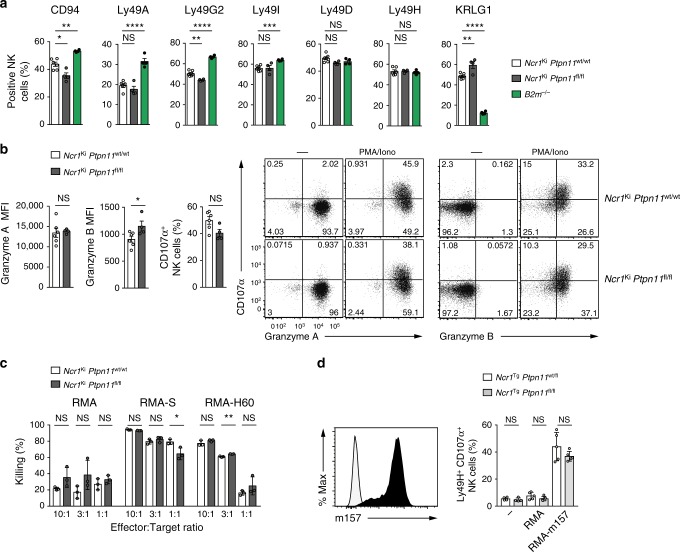
Shp-2 is largely dispensable for NK cell effector functions. **a** Graphs depict percentages of CD94^+^, Ly49A^+^, Ly49G2^+^, Ly49I^+^, Ly49D^+^, Ly49H^+^, and KLRG1^+^ splenic NK cells (gated as NK1.1^+^ CD3/CD19^−^) from *Ncr1*^Ki^ *Ptpn11*^wt/wt^ (white), *Ncr1*^Ki^ *Ptpn11*^fl/fl^ (dark gray), and *B2m*^*−/−*^ (green) mice. **b** Graph and a representative cytometric plot illustrate the production of granzyme A and B by splenic NK cells (gated as NK1.1^+^NKp46^+^CD3/CD19^−^) after phorbol 12-myristate 13-acetate and ionomycin stimulation (PMA/Iono). **c** NK cells isolated from polyinosinic:polycytidylic acid (polyI:C)-treated *Ncr1*^Ki^ *Ptpn11*^wt/wt^ or *Ncr1*^Ki^ *Ptpn11*^fl/fl^ mice were plated with RMA, RMA-S, or RMA-H60 cells at the indicated ratios. The graph depicts percentage killing of target cells, as measured by quantifying PI^−^ living target cells. **d** Naive splenocytes from *Ncr1*^Tg^ *Ptpn11*^fl/fl^ (light gray) mice or heterozygote littermate controls (white) were incubated with RMA cells or RMA-m157 cells (expression of m157 is illustrated in the graph on the left). Percentage of YFP^+^Ly49H^+^CD107α^+^ NK cells in each group was determined by flow cytometry (illustrated in the graph on the right). **a**, **b** Results represent the mean ± SEM of *n* = 4 (*Ncr1*^Ki^ *Ptpn11*^fl/fl^ or *B2m*^*−/−*^) and *n* = 6 (*Ncr1*^Ki^ *Ptpn11*^wt/wt^) mice per genotype and are representative of at least three (**a**) and two (**b**) independent experiments. **c**, **d** Results represent the mean ± SD of *n* = 3 (**c**), *n* = 4 (for non-stimulated conditions) and *n* = 5 (for RMA conditions) (**d**) technical replicates and are representative of at least two (**c**) and three (**d**) independent experiments. Statistical comparisons are shown; *p ≤ 0.05, **p ≤ 0.01, ***p ≤ 0.001, ****p ≤ 0.0001, NS, non-significant; Student’s *t*-test. Source data are provided as a Source Data file

Because inhibitory receptors expressed on NK cells have been shown to recruit Shp-2, we investigated the education state of NK cells lacking this phosphatase^21,22^. We first characterized the receptor repertoire, which exhibits increased levels of Ly49 inhibitory receptors and decreased expression of KLRG1 in non-educated NK cells, as exemplified by NK cells derived from *β2* *m*^*−/−*^ mice (Fig. 2a). However, Shp-2 deficiency did not cause such changes, allowing expression of a largely normal repertoire (Fig. 2a).

To assess the role of Shp-2 in NK cell effector functions, we first measured their ability to produce cytokines and cytotoxic mediators in response to phorbol 12-myristate 13-acetate (PMA)/ionomycin stimulation. No defects in Granzyme A (GzmA), GzmB, or degranulation (quantified by surface CD107α) were observed (Fig. 2b). We then examined the effector response to “missing-self” targets and induced by engagement of two well characterized NK cell activating receptors, NKG2D and Ly49H. We performed an in vitro killing assay using RMA cells (H-2^b^) and variants lacking MHC class I (RMA-S), or bearing the NKG2D ligand H60 (RMA-H60^26^) as targets. We found that Shp-2*-*deficient and wild-type in vivo primed NK cells killed RMA-S and RMA-H60 to comparable extents (Fig. 2c). Using RMA cells transfected with the Ly49H ligand M157, we observed that Shp-2-deficient and wild-type Ly49H^+^ NK cells exhibited comparable degranulation levels (Fig. 2d). Taken together, these data demonstrate that the absence of Shp-2 does not impair NK cell effector functions. This contrasts with the hyporesponsive phenotype of both SHIP-1 and Shp-1 deficient NK cells^7–11^.

### Role of Shp-2 in response to IL-15

**Fig. 3:**
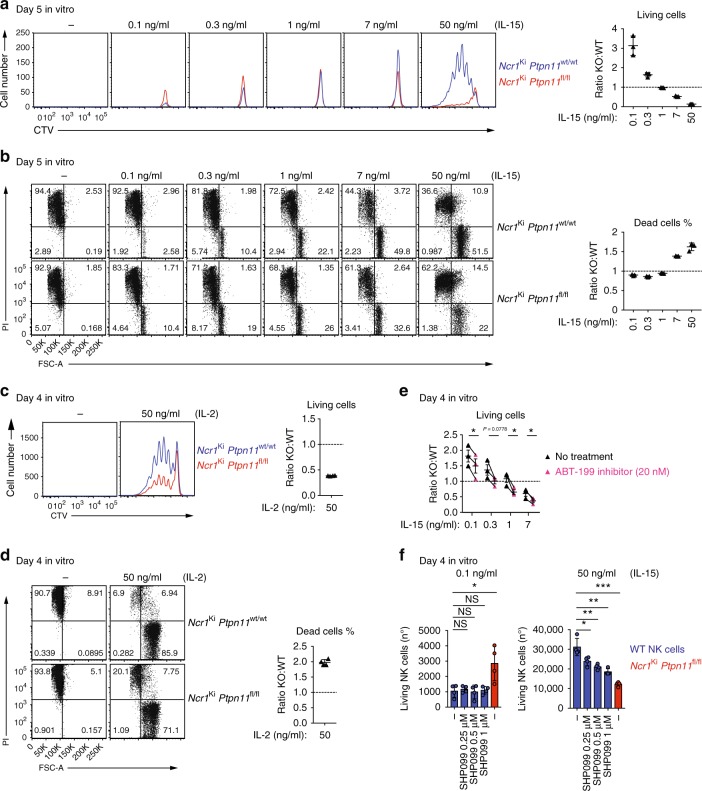
*Ptpn11*-deficient NK cells show a biphasic response to IL-15. **a**–**d** Enriched NK cells from *Ncr1*^Ki^ *Ptpn11*^wt/wt^ (blue; congenically marked) and *Ncr1*^Ki^ *Ptpn11*^fl/fl^ (red) mice were co-cultured for 5 days in the presence of the indicated amounts of IL-15 (**a**, **b**) or were co-cultured for 4 days with 50 ng/ml of IL-2 (**c**, **d**). **a**, **c** Histograms represent the amount and cell division (CTV dilution) of Shp-2-deficient and control NK living cells in each condition (quantitative flow cytometry acquisition) and ratios thereof. **b**, **d** Representative flow cytometry plots show the percentage of dead cells (PI^+^) among knockout and control NK cells and ratios thereof. **e** Enriched Shp-2-deficient and congenically marked control NK cells were co-cultured for 4 days in the presence of the indicated amounts of IL-15 and 0 (black triangles) or 20 nM ABT-199 (pink triangles). The graph depicts the ratio of living knockout over control NK cells. **f** Enriched NK cells from C57BL/6 wild-type mice (blue), or Shp-2-deficient NK cells from *Ncr1*^Ki^ *Ptpn11*^fl/fl^ mice (red) as control, were cultured for 4 days in the presence of 0.1 or 50 ng/ml IL-15 and 0, 0.25, 0.5, or 1 μM SHP099. Graphs depict the number of living (PI^−^) NK cells. Results represent the mean ± SD of *n* = 3 (**a**, **b**) and *n* = 4 (**c**, **d**, **f**) replicates, or of *n* = 3 independent experiments (**e**; average of each condition/experiment). Results are representative of at least five (**a**, **c**), two (**b**, **d**), or three (**e**, **f**) independent experiments. Statistical comparisons are shown in (**e**), (**f**); NS, non-significant, *p ≤ 0.05, **p ≤ 0.01, ***p ≤ 0.001; Student’s *t*-test unpaired (**f**) and Student’s *t*-test paired (**e**). Source data are provided as a Source Data file

Our finding that Shp-2-deficient mature NK cells were increased in numbers as compared with control NK cells led us to investigate their response to IL-15, which is essential for NK cell proliferation and survival^27^. We first assessed the expression of the IL-15 receptor (IL-15R) chains on *Ptpn11*-deficient and control NK cells^28^. Both common γ subunit (γc; CD132) and IL-15Rβ chain (CD122) were expressed at normal levels, ruling out a defect of IL-15 receptor cell surface expression (Supplementary Fig. 2a and b). We next investigated whether the response of Shp-2-deficient NK cells to IL-15 was altered. At low IL-15 concentration Shp-2-deficient NK cells presented a survival advantage, as shown by the increased number of recovered cells (Fig. 3a) and the lower percentage of dying cells (propidium iodide (PI)^+^; Fig. 3b). Conversely, high IL-15 concentration was detrimental to these cells, as observed both at 7 ng/ml, in the absence of cell division, as well as at 50 ng/ml, in the presence of proliferation, as shown by cell trace Violet (CTV) and PI staining (Fig. 3a, b). Notably, the defective expansion of NK cells was also observed in response to high doses IL-2 (Fig. 3c, d).

The survival advantage at low IL-15 doses was consistent with the accumulation of Shp-2-deficient NK cells observed under physiological conditions in vivo (Fig. 1). Given the important role of the antiapoptotic protein B cell leukemia/lymphoma 2 (Bcl-2) in sustaining survival of resting NK lymphocytes^29^, we next determined its expression level in knockout and control NK cells. We found that Shp-2 deficient NK cells expressed higher Bcl-2 levels than control NK cells (Supplementary Fig. 2c). Interestingly, while Bcl-2 expression decreased throughout maturation of wild-type NK cells, it remained constant in Shp-2-deficient NK lymphocytes, in agreement with the increased numbers of the CD11b SP subset (Supplementary Fig. 2d). To validate these findings, we assessed the effects of the Bcl-2 inhibitor ABT-199 on survival of co-cultured Shp-2-deficient and control NK cells. Bcl-2 inhibitor treatment preferentially affected the survival of the former, as shown by the decrease of the ratio of living Shp-2-deficient over control NK cells (Fig. 3e). Furthermore, inhibition of myeloid cell leukemia sequence 1 (Mcl1), a second major antiapoptotic protein, did not revert the survival advantage of Shp-2-deficient cells (Supplementary Fig. 2e) at low IL-15 concentrations, underscoring the contribution of Bcl-2.

We next wondered whether the effects of Shp-2 deficiency in presence of low and high IL-15 concentrations were recapitulated by supplying the Shp-2 inhibitor SHP099 to wild-type NK cell cultures^20^. While SHP099 decreased NK cell expansion at high IL-15 doses, it did not increase their survival in the presence of low IL-15 concentrations (Fig. 3f). This suggests that the survival advantage of Shp-2-knockout NK cells reflects an adapted state of these cells to their defective condition in vivo, rather than a direct effect of Shp-2 activity, and we thus focused on the effects at high doses.

### Shp-2 supports features linked to NK cell activation

**Fig. 4:**
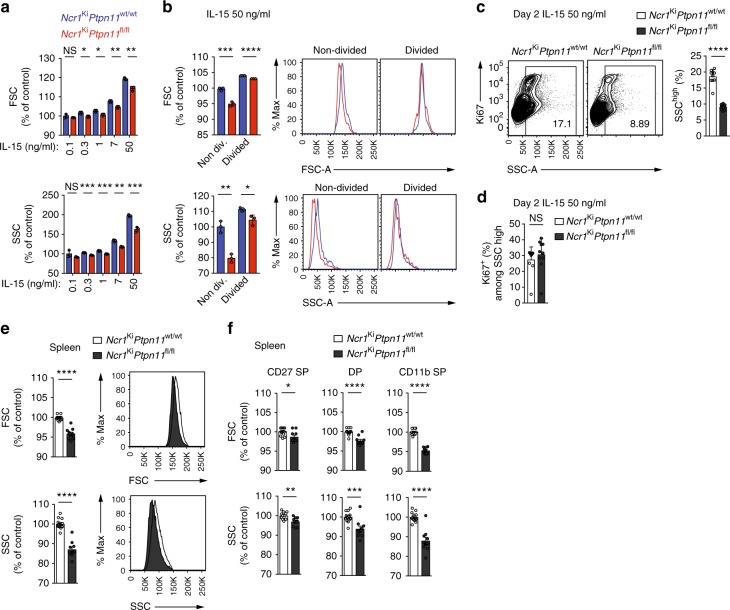
Shp-2-deficiency alters NK cell size and granularity. **a**, **b** Enriched NK cells from *Ncr1*^Ki^ *Ptpn11*^wt/wt^ (blue; congenically marked) and *Ncr1*^Ki^ *Ptpn11*^fl/fl^ (red) mice were co-cultured for 5 days in the presence of the indicated amounts of IL-15. **a** Quantification of the geometric mean of forward scatter (FSC) and side scatter (SSC) of knockout and control NK cells (the average of the NK cells in culture with 0.1 ng/mL of IL-15 from *Ncr1*^Ki^ *Ptpn11*^wt/wt^ mice was set as 100%). **b** Quantification and representative flow cytometry plots depicting the geometric mean of FSC and SSC of non-divided and divided NK cells (the average of the non-divided NK cells from *Ncr1*^Ki^ *Ptpn11*^wt/wt^ mice was set as 100%). **c**, **d** Enriched NK cells from *Ncr1*^Ki^ *Ptpn11*^wt/wt^ (white) and *Ncr1*^Ki^ *Ptpn11*^fl/fl^ (dark gray) mice were co-cultured for 2 days in the presence of 50 ng/ml of IL-15. Representative flow cytometry plots illustrating Ki67 and SSC and a quantification of the percentage of SSC^high^ cells (**c**) and of Ki67^+^ among SSC^high^ (**d**) non-divided control and knockout NK cells (gated as NK1.1^+^CD3/CD19^−^ and cell trace violet (CTV) high). **e**, **f** Representative cytometric profile and quantification of FSC and SSC for total (**e**), CD27 SP, DP, and CD11b SP NK cell populations (**f**) in the spleen (gated as NK1.1^+^NKp46^+^CD3/CD19^−^). For quantification, the average of *Ncr1*^Ki^ *Ptpn11*^wt/wt^ was set as 100%. Results represent the mean ± SD of *n* = 3 (**a**, **b**) and *n* = 10 (**c**, **d**) technical replicates per genotype and are representative of at least two independent experiments (**a**, **b**) or are a pool of two independent experiments (**c**, **d**). Results represent the mean ± SEM of *n* = 10 (*Ncr1*^Ki^ *Ptpn11*^fl/fl^) and *n* = 13 (*Ncr1*^Ki^ *Ptpn11*^wt/wt^) mice (**e**, **f**) per genotype and are a pool of three independent experiments (**e**, **f**). Statistical comparisons are shown; *p ≤ 0.05, **p ≤ 0.01, ***p ≤ 0.001, ****p ≤ 0.0001, NS, non-significant; Student’s *t*-test. Source data are provided as a Source Data file

Our findings that the survival and expansion of Shp-2-deficient NK cell were impaired at high IL-15 doses (Fig. 3a, b) led us to investigate whether the absence of Shp-2 affected NK cell activation by acute IL-15 exposure. Under these conditions, NK lymphocytes acquire a blastoid morphology with increased granularity. We thus examined the morphology of Shp-2-deficient and control NK cells cultured in the presence of various IL-15 doses. Although the size and, in particular, the granularity of Shp-2-deficient NK cells augmented in response to IL-15 (Fig. 4a), they increased significantly less than in wild-type NK cells. This difference was mainly due to non-divided Shp-2-deficient cells (Fig. 4b), suggesting that cell division occurred after reaching a given size and granularity. To better understand this phenomenon, we assessed cell cycle entry and granularity when cells are starting to divide. The proliferation marker Ki67 was expressed in highly granular cells (Fig. 4c). Furthermore, highly granular, non-divided Shp-2-deficient NK cells were significantly reduced as compared with the control counterparts (Fig. 4c), but not the proportion of Ki67^+^ NK cells among them (Fig. 4d). This indicates that, in Shp-2-deficient cells, the division defect in response to IL-15 correlates with the granularity defect.

These results prompted us to analyze size and granularity of Shp-2-deficient NK cells under physiological conditions in naive animals. We found that NK cells from BM and spleen of *Ncr1*^Ki^ *Ptpn11*^fl/fl^ mice were significantly affected in both parameters (Supplementary Fig. 3a and Fig. 4e), especially in the CD11b SP NK cell stage (Supplementary Fig. 3b and Fig. 4f). This phenotype was also observed in splenic CD11b SP NK cells from the *Ncr1*^Tg^ *Ptpn11*^fl/fl^ line (Supplementary Fig. 3c and d). Importantly, the defect was cell-intrinsic as the decreased size and granularity was conserved in Shp-2-deficient NK cells from mixed BM chimeric mice (Supplementary Fig. 3e and f). Altogether, these results suggest a role for Shp-2 in NK cell metabolism, alike what has been established in a variety of non-immune tissues.

### Shp-2 mediates metabolic rewiring in response to high IL-15

**Fig. 5:**
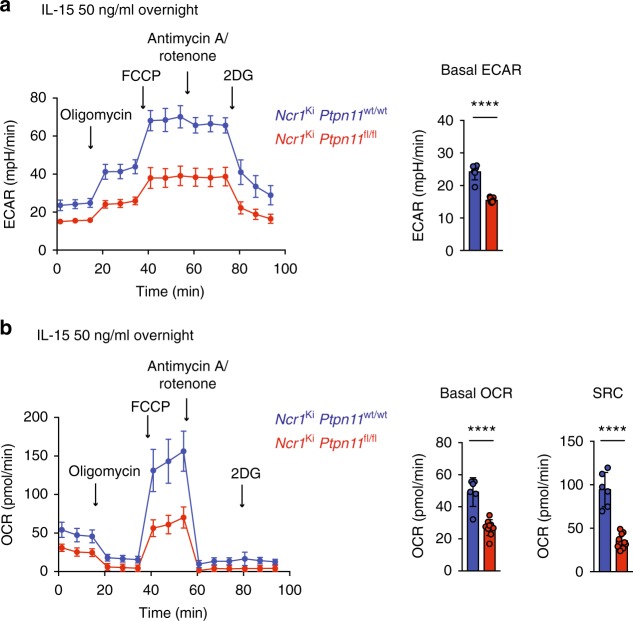
*Ptpn11*-deficient NK cells show a defective energetic response to IL-15. **a**, **b** Extracellular acidification rate (ECAR, **a**) and oxygen consumption rates (OCR, **b**) prior and after addition of the indicated mitochondrial inhibitors (oligomycin, trifluoromethoxy carbonylcyanide phenylhydrazone (FCCP), antimycin A, rotenone, and 2-deoxy-D-glucose (2DG) were measured for NK cells from *Ncr1*^Ki^ *Ptpn11*^wt/wt^ (blue) and *Ncr1*^Ki^ *Ptpn11*^fl/fl^ (red) mice cultured overnight in the presence of IL-15 (50 ng/ml). SRC (spare respiratory capacity). Results represent the mean ± SD of *n* = 6 (*Ncr1*^Ki^ *Ptpn11*^wt/wt^) and *n* = 9 (*Ncr1*^Ki^ *Ptpn11*^fl/fl^) replicates per genotype and are representative of at least three independent experiments. Statistical comparisons are shown on bar graphs; ****p ≤ 0.0001; Student’s *t*-test. Source data are provided as a Source Data file

Similar to other immune cells, metabolic activation was recently revealed to be a critical event in NK cell activation, proliferation as well as survival^30^. The results described above led us to hypothesize that the expression of Shp-2 might orchestrate proper NK cell metabolic activity following exposure to IL-15. By examining cellular metabolic activity with Seahorse technology, we found that basal and maximal glycolytic rate of Shp-2-deficient NK cells (as shown by extracellular acidification) were reduced (Fig. 5a). This suggested that Shp-2-deficient NK cells failed to engage aerobic glycolysis to support their expansion. In addition, Shp-2-deficient NK cells also displayed reduced basal oxygen consumption and spare respiratory capacity as measured by oxygen consumption rates (OCR) (Fig. 5b), indicating that they might lack the ability to adjust mitochondrial activity for energy production in response to metabolic stress. Taken together, these results demonstrate that Shp-2-deficient NK cells were unable to properly raise both glycolytic and oxidative phosphorylation capacities in response to IL-15, which may contribute to their phenotypic defects.

### Shp-2 is a key mediator of IL-15-induced ERK activation

**Fig. 6:**
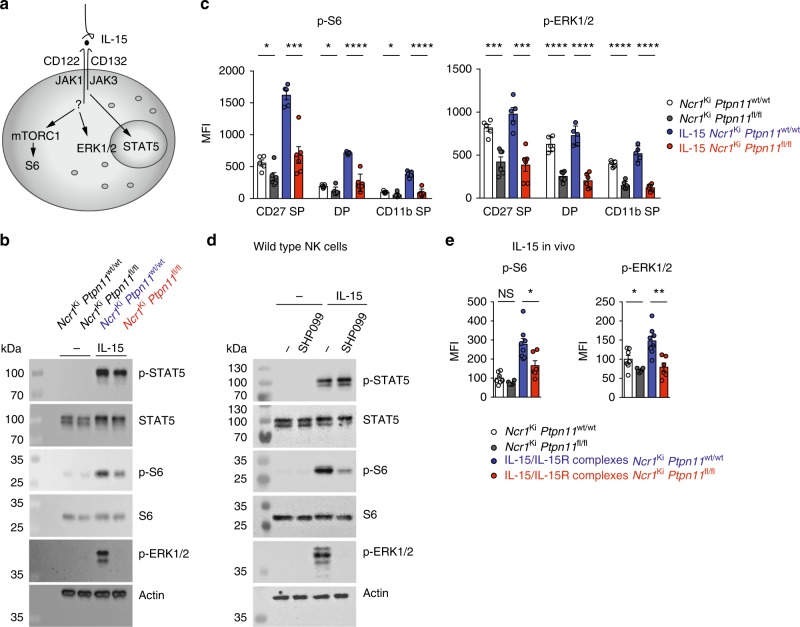
*Ptpn11*-deficient NK cells have a curtailed response to IL-15. **a** Schematic representation of the main signaling pathways engaged downstream of IL-15R in NK cells. **b**, **c** Purified NK cells (**b**) or splenocytes (**c**) from *Ncr1*^Ki^ *Ptpn11*^wt/wt^ and *Ncr1*^Ki^ *Ptpn11*^fl/fl^ mice were cultured in vitro for 30 min in the presence of 50 ng/ml IL-15 (blue and red, respectively) or left untreated (white and dark gray, respectively). Phosphorylation of STAT5, ERK, and S6 were measured by immunoblot analysis (**b**) and by flow cytometry in CD27 SP, DP, and CD11b SP NK cell subsets (gated as NK1.1^+^ and CD3/CD19^−^) (**c**). **d** Purified NK cells from C57BL/6 wild-type mice were cultured in vitro for 30 min in the presence of 50 ng/ml IL-15 or left untreated and in the presence of 4 μM SHP099. Phosphorylation of the indicated proteins was measured by immunoblot analysis. Actin was used as loading control (**b**, **d**). **e** Phosphorylation of ERK and S6 (expressed as geometric MFI) on splenic NK cells of untreated mice or of mice treated with IL-15/IL-15R complexes 1 day prior to the analysis are illustrated. Results represent the mean ± SEM of *n* = 5 (*Ncr1*^Ki^ *Ptpn11*^wt/wt^) and *n* = 6 (*Ncr1*^Ki^ *Ptpn11*^fl/fl^) (**c**), *n* = 6 (*Ncr1*^Ki^ *Ptpn11*^fl/fl^) and *n* = 7 or *n* = 8 (*Ncr1*^Ki^ *Ptpn11*^wt/wt^) (**e**) mice per genotype and are a pool of two independent experiments (**e**) or representative of at least two independent experiments (**b**–**d**). Statistical comparisons are shown (**c**, **e**); *p ≤ 0.05, **p ≤ 0.01, ***p ≤ 0.001, ****p ≤ 0.0001, NS, non-significant; Student’s *t*-test. Source data are provided as a Source Data file

To gain insight into the molecular mechanisms leading to the altered IL-15 response of Shp-2-deficient NK cells, we investigated the different known IL-15-dependent signaling pathways. These include the engagement of Janus kinase (Jak)-Signal transducer and activator of transcription (STAT) 5, mTOR, and extracellular signal-regulated kinases (ERKs) (Fig. 6a)^27,30–34^. Whereas Shp-2-deficient NK cells had nearly normal levels of STAT5 phosphorylation after 30 min of IL-15 stimulation, phosphorylation of S6, a target of mTOR complex 1 (mTORC1)^30,33–36^, was strongly decreased while phosphorylation of ERK was abrogated (Fig. 6b). To ensure that the results were not biased by the different composition of the NK cell maturation subsets, we assessed their respective phosphorylation status of S6 and ERK by flow cytometry. We found that signaling defects were present in all NK cells, irrespective of the maturation stage (Fig. 6c). Similar signaling defects were witnessed after 1 h of IL-15 stimulation, with the exception of ERK, whose induction is too weak to be robustly detected (Supplementary Fig. 4a). Importantly, Shp-2-deficient NK cells exhibited lower basal levels of S6 and ERK phosphorylation, indicating that Shp-2 constitutively contributed to these pathways, likely downstream of endogenous IL-15 (Fig. 6c).

In order to exclude indirect effects of Shp-2 deficiency, we assessed phosphorylation of S6 and ERK following stimulation with PMA/ionomycin, which bypasses receptor proximal signaling. We observed normal engagement of these pathways in Shp-2-deficient NK cells (Supplementary Fig. 4b). We next stimulated wild-type NK cells with IL-15 in the presence or absence of the Shp-2 inhibitor SHP099. In agreement with the genetic approach, we observed a virtually normal engagement of the Jak-STAT5 pathway, but a severe defect in ERK and S6 phosphorylation (Fig. 6d). To corroborate the in vitro results, we assessed the in vivo role of Shp-2 in response to stimulation with IL-15/IL-15R complexes^37^. One-day post-treatment, we evaluated IL-15 signaling in NK cells ex vivo. In agreement with the in vitro studies, we found that control NK cells presented prominent phosphorylation of ERK and S6, while their Shp-2-deleted counterparts displayed a strong defect in these signaling pathways (Fig. 6e). Taken together, these results demonstrate that—downstream of IL-15R—Shp-2 is required for ERK and, presumably, mTORC1 engagement^30,33–36^.

### mTOR and ERK contribute to IL-15-driven NK cell expansion

**Fig. 7:**
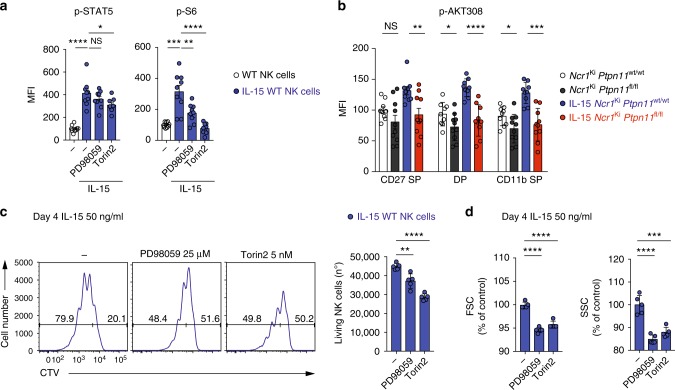
mTOR and ERK inhibition phenocopy the expansion defect of Shp-2-deficient NK cells. **a** Splenocytes from C57BL/6 wild-type mice were cultured in vitro for 30 min in the presence of 50 ng/ml IL-15 (blue) and in the presence of 50 µM PD98059 or 250 nM Torin2, or left untreated (white). Phosphorylation of the indicated proteins was measured by flow cytometry. **b** Splenocytes from *Ncr1*^Ki^ *Ptpn11*^wt/wt^ and *Ncr1*^Ki^ *Ptpn11*^fl/fl^ mice were cultured in vitro for 30 min in the presence of 50 ng/ml IL-15 (blue and red, respectively) or left untreated (white and dark gray, respectively). Phosphorylation of Akt (position 308) was measured by flow cytometry in CD27 SP, DP, and CD11b SP NK cell subsets (gated as NK1.1^+^ and CD3/CD19^−^). For quantification, the average of the untreated NK cells from *Ncr1*^Ki^ *Ptpn11*^wt/wt^ mice was set as 100%. **c**, **d** NK cells from C57BL/6 wild-type mice were cultured in vitro with 50 ng/ml IL-15 for 4 days; PD98059 was added daily (day 0, 1, 2, and 3) at a concentration of 25 μM, while Torin2 was added once (day 0) at a concentration of 5 nM. **c** Histograms represent the amount and cell division (CTV dilution) of living NK cells in each condition (quantitative flow cytometry acquisition) and graph the number of living NK cells (gated as propidium iodide (PI)^–^NK1.1^+^CD3/CD19^–^) after 4 days of culture for each condition. **d** Graphs depict the geometric mean of FSC and SSC of non-divided NK cells across conditions (gated as PI^–^NK1.1^+^CD3/CD19^−^ and cell trace violet (CTV) high). For quantification, the average of IL-15-expanded NK cells was set as 100%. Results represent the mean ± SEM of *n* = 9 (**a**), *n* = 9 (*Ncr1*^Ki^ *Ptpn11*^wt/wt^) and *n* = 10 mice (*Ncr1*^Ki^ *Ptpn11*^fl/fl^) (**b**) and are a pool of two independent experiments (**a**, **b**). Results represent the mean ± SD of *n* = 5 technical replicates and are representative of two independent experiments (**c**, **d**). Statistical comparisons are shown (**a**–**d**); *p ≤ 0.05, **p ≤ 0.01, ***p ≤ 0.001, ****p ≤ 0.0001, NS, non-significant; Student’s *t*-test. Source data are provided as a Source Data file

We next wondered whether the effect of Shp-2 on S6 phosphorylation is mediated by ERK. ERK has been reported to suppress the mTORC1 inhibitor tuberous sclerosis complex (TSC), thereby activating the mTORC1 pathway, and can further regulate S6 phosphorylation through 90 kDa ribosomal S6 kinase^38–40^. We therefore assessed the effects of the mitogen-activated protein kinase kinase (MKK/MEK) inhibitor PD98059 on S6 phosphorylation. PD98059 partially reduced S6 phosphorylation induced by IL-15 exposure, while it had no effect on STAT5 (Fig. 7a). Importantly, in agreement with previous findings^30^, Torin2, an inhibitor of mTORC1 and mTORC2, completely prevented S6 phosphorylation (Fig. 7a). We also assessed the phosphorylation of Akt at position 308, which is regulated by PI3K and contributes to Akt-mediated inactivation of the TSC complex and the subsequent mTORC1 activation^41^. Interestingly, we found that Akt phosphorylation at position 308 was largely dependent on Shp-2 after 30 min and 1 h of stimulation (Fig. 7b and Supplementary Fig. 5a). Altogether, these data suggest that Shp-2 regulates PI3K-Akt and ERK engagement, which might further contribute to mTORC1 and S6 activation (Supplementary Fig. 5b).

To test whether defective ERK and mTORC1 signaling in Shp-2-deficient cells account for the proliferation and the survival defects observed in response to high IL-15 (Fig. 3a, b and Fig. 4c, d), we cultured wild-type NK cells for 4 days in the presence of PD98059 or Torin2. These inhibitors were supplied at low concentrations in order to minimize aspecific effects. Reducing the activity of ERK and, most prominently, mTOR led to decreased proliferation and lower numbers of living NK cells (Fig. 7c). In addition, a reduction of NK cell size and granularity was observed (Fig. 7d). Taken together, these data support the hypothesis that signaling by mTOR and ERK contribute to the effects of Shp-2 in response to IL-15.

### Loss of Shp-2 affects MCMV-induced NK cell expansion

**Fig. 8:**
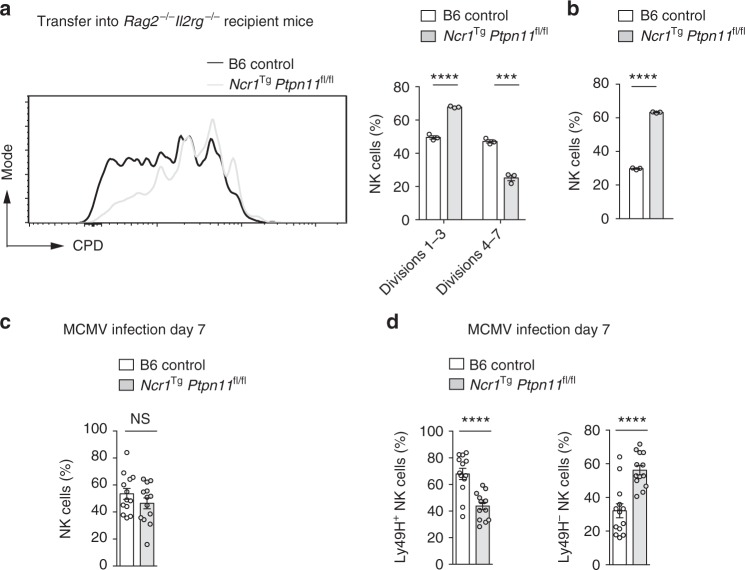
Shp-2 mediates NK cell activation in response to IL-15 in vivo. **a**, **b** CPD-labeled splenic YFP^+^ NK cells from *Ncr1*^Tg^ *Ptpn11*^fl/fl^ (CD45.2^+^; light gray line and bars) mice were sorted and injected at a 1:1 ratio with sorted splenic NK cells from B6.SJL (CD45.1^+^; black line and white bars) congenic mice into recipient *Rag2*^*−/−*^*Il2Rg*^*−/−*^ mice. Percentage of cells in divisions 1–3 or 4–7 (**a**) and relative proportion of total NK cells were determined at day 4 post-transfer (**b**). **c**, **d** Splenic YFP^+^ NK cells from *Ncr1*^Tg^ *Ptpn11*^fl/fl^ROSA^EYFP^ (CD45.2^+^) mice were sorted and injected at a 1:1 ratio with sorted splenic NK cells from B6.SJL (CD45.1^+^) congenic mice into MCMV infected NK cell-deficient mice (*Ncr1cre*^*Ki*^ROSA-DTA). Frequencies of total NK cells (**c**), and of Ly49H^+^ and Ly49H^−^ NK cells (**d**) were determined at day 7 post-infection. Results represent mean ± SEM of *n* = 3 mice (**a**, **b**) or of *n* = 13 mice (**c**, **d**), and are representative of three independent experiments (**a**, **b**) or a pool of three independent experiments (**c**, **d**). Statistical comparisons are shown; ***p ≤ 0.001, ****p ≤ 0.0001, NS, non-significant; Student’s *t*-test. Source data are provided as a Source Data file

To study the effects of endogenous IL-15 in vivo, we examined NK cell homeostatic proliferation following adoptive transfer into lymphopenic animals^42,43^. CPD-labeled NK cells isolated from *Ncr1*^Tg^ *Ptpn11*^fl/fl^ were mixed at a 1:1 ratio with congenically marked B6.SJL NK cells and co-transferred into *Rag2*^*−/−*^*Il2Rg*^*−/−*^ recipients. We found that wild-type NK cells divided significantly more than Shp-2-deficient NK cells (Fig. 8a), confirming an essential role of Shp-2 in IL-15-driven proliferation in vivo. However, despite their impaired division, Shp-2-deficient NK cells were overrepresented (Fig. 8b), consistent with their survival advantage at low IL-15 doses (Fig. 3) and increased numbers at steady state in vivo (Fig. 1).

To dissect Shp-2 contrasting roles in a more defined and physiologically relevant infectious setting, we took advantage of the well characterized MCMV model^44–46^. In this model, engagement of the activating receptor Ly49H by the MCMV protein m157 leads to preferential expansion of Ly49H^+^ NK cells, as Ly49H signaling amplifies the response to IL-15 (Supplementary Fig. 6a)^44^. We reasoned that the absence of Shp-2 may be disadvantageous for the Ly49H^+^ NK cells, but might improve the survival of Ly49H^−^ NK cells. To test this hypothesis, NK cells isolated from *Ncr1*^Tg^ *Ptpn11*^fl/fl^ were mixed at a 1:1 ratio with B6.SJL NK and transferred into NK cell-deficient mice (*Ncr1cre*^Ki^ROSA-DTA mice), which were simultaneously infected with MCMV. Seven days post-infection, Shp-2-deficient and control donor NK cells were found at the same ratio (Fig. 8c). However, wild-type NK cells were overrepresented in the Ly49H^+^ subset, while Shp-2-deficient NK cells in the Ly49H^−^ subset (Fig. 8d). These data illustrate the importance of Shp-2 in calibrating IL-15-induced signaling to optimize a specific NK cell response during viral infection.

## Discussion

The effects of Shp-2 on NK cell development and functions have not been defined. Using two different mouse lines conditionally deficient for Shp-2 in the NK lineage, we found that NK cell development appeared for the most part normal, with the exception of an increased number of the most mature NK cells. Unexpectedly, we also observed that NK cell effector functions were not impaired in these animals. Shp-2 binding to phosphorylated ITIMs has been well documented, in particular in the context of T-cell inhibitory receptors^21,22,47–49^. However, its in vivo function in both NK and T cells is less clear. In fact, we recently reported that genetic deletion of *Ptpn11* did not revert the dysfunctional state of exhausted T cells^50^. Altogether, these data suggest that redundant mechanisms compensate for Shp-2 absence and that Shp-2 fulfills other functions in these cells.

The importance of Shp-2 in controlling the development of several organs and tissues by relaying signaling from growth factor receptors to MAPKs is well-established^12,17,51^. In NK cells, activation of this pathway, as well as metabolism and proliferation are tightly regulated by selected cytokines, especially IL-15^27,30–33^. We therefore investigated whether Shp-2 regulated the NK cell response to IL-15. Notably, we found that, in the absence of Shp-2, IL-15-induced MAPK signaling was abolished. Our data further suggest that Shp-2 is important for metabolic activation through regulation of the PI3K-Akt-mTORC1 axis, although the relevance of the Akt-mTORC1 connection in NK cells remains to be clarified^35,41^, or indirectly through its effects on ERK.

Over a decade ago, recruitment of Shp-2 to the IL-15 receptor was shown in response to IL-2 and IL-15 in both T and NK cells and a positive role for Shp-2 in ERK activation was observed downstream of IL-2 receptor in fibroblasts^52–55^, supporting our data on the importance of Shp-2 downstream of the IL-15 and IL-2 receptors. However, Shp-2 deficiency does not phenocopy the NK cell defects observed in IL-15- and PI3K-deficient mice or in mice conditionally deficient for mTORC1 and mTORC2 in the NK lineage^30,34,43,56^. In these mouse models, NK cells present severe developmental, maturation, and numerical defects, while Shp-2 deficiency leads to rather increased numbers of the most mature NK cells. Regarding the mechanism, we have unveiled that Shp-2 uncouples the Jak-STAT5 from the ERK pathway and future investigations are required to more precisely define the intersection of Shp-2 with the totality of the signaling cascades induced by IL-15.

It has been recently reported that constitutive metabolic overactivation is detrimental for NK cell development and survival in vivo^57,58^. NK cells lacking Tsc1 exhibited an overactive phenotype and underwent apoptosis^57^. In addition, NK cells deficient for the transcription factor Rfx7 presented an increased metabolism and were preferentially lost at physiological/limiting cytokine concentrations, while had a survival advantage at high IL-15 doses^58^. These findings mirror the results described here, showing that the lowered metabolic state of Shp-2-deficient NK cell helps their maintenance and accumulation both in vivo and at low IL-15 concentrations in vitro, while is unfavorable at high IL-15 doses. In line with these results, NK cell responses to low IL-15 doses are dominated by the Jak-STAT5 pathway, which is nearly normal in the absence of Shp-2^30^. Similarly to NK cells deficient for 3-phosphoinositide-dependent kinase 1 (PDK1), an upstream activator of mTORC1, Shp-2-deficient NK cells express higher levels of Bcl-2, which supports their survival^29,41,59^. This antiapoptotic protein was particularly abundant in the CD11b SP subset, strongly correlating with their accumulation. However, the increased survival at limiting IL-15 doses was not recapitulated by in vitro treatment of wild-type NK cells with the Shp-2 inhibitor. This discrepancy could be explained by broader effects of the inhibitor or suggest that this phenotype reflects an adaptation occurring during development in vivo, a point that might be relevant with respect to long-term treatment with Shp-2 inhibitors.

Not surprisingly, given the impaired signaling pathways, Shp-2 deletion affected the metabolic reprogramming of NK cells, which presented lower glycolysis, oxidative phosphorylation, as well as spared respiratory capacity following IL-15 exposure. As a consequence, Shp-2 deficient NK cells failed to increase their cellular mass and granularity and did not divide at high IL-15 doses. Importantly, this expansion defect was recapitulated upon IL-2 stimulation and during MCMV infection in Ly49H^+^ NK cells, which have augmented sensitivity to IL-15^44^.

Altogether, our data demonstrate that Shp-2 is critical in the context of acute IL-15 stimulation and in the presence of an activating co-stimulus in vivo. We therefore propose that Shp-2 main function in NK cells is not to calibrate the threshold of NK cell reactivity but instead to mediate IL-15 signaling, actively favoring specific NK cell responses. A growing number of clinical studies aim to harness NK cell anti-tumor immunity, while selective Shp-2 inhibitors have been developed for treatment of cancers relying on this pathway^60^. Based on our data, we argue that therapeutic approaches combining Shp-2 inhibition with NK cell stimulating regimens should be reevaluated and possibly avoided. The use of this inhibitor might instead be beneficial in the management of the aggressive large granular lymphocyte leukemia, which has been proposed to depend on IL-15^61–63^.

## Methods

### Mice

NK cell-specific knockout mice for *Ptpn11* were generated by crossing either the *Ptpn11*^fl/fl^ mice^15^ to the *Ncr1cre*^Ki^ deleter strain^23^ or the *Ptpn11*^fl/fl^ mice^24^ to the *Ncr1cre*^Tg^ mice^64^, generating *Ncr1*^Ki^*Ptpn11*^fl/fl^ and *Ncr1*^Tg^ *Ptpn11*^fl/fl^, respectively. *Ncr1cre*^Tg^ *Ptpn11*^fl/fl^ mice were crossed to R26R-EFYP mice to monitor cre activity.

For genotyping *Ncr1*^Ki^*Ptpn11*^fl/fl^ mice, *Ptpn11*-floxed alleles were detected by PCR amplification using a set of two primers that amplify a 300 bp wt band and a 380 bp floxed band (forward primer 5′-*ATGACTCCTGAAGCCCATTG*-3′ and reverse primer 5′-*TTCCCATCACCTCAGACTCC*-3′). For genotyping *Ncr1*^Tg^ *Ptpn11*^fl/fl^ mice, *Ptpn11*-floxed alleles were detected by PCR amplification using a set of two primers that amplify a 312 bp wt band and a 430 bp floxed band (forward primer 5′-*TAGCTGCTTTAACCCTCTGTGT*-3′; reverse primer 5′-*CATCAGAGCAGGCCATATTCC*-3′).

*Ptpn11*^fl/fl15^, *Ncr1cre*^Ki^, B6.SJL, *β2m*^−/−^, and C57BL/6 mice, all on a C57BL/6 (H2^b^) background, were bred under specific pathogen-free conditions at the animal facility of the University of Lausanne. Sex- and age-matched 6–14-week-old mice were used for the experiments. All animal experimental protocols were approved by the Veterinary office regulations of the State of Vaud, Switzerland, and all methods were performed in accordance with the Swiss guidelines and regulations.

C57BL/6, B6.SJL (B6.SJL-Ptprca Pepcb/BoyJ), R26R-EFYP (B6.129×1 *Gt(ROSA)26Sor*^*tm1(EYFP)Cos*^/J), and ROSA-DTA (B6.129P2-Gt(ROSA)26Sortm1(DTA)Lky/J) mice were purchased from The Jackson Laboratory (Bar Harbor, ME). *Rag2*^*−/−*^*Il-2Rg*^*−/−*^ (B10;B6-*Rag2*^*tm1Fwa*^ *Il2rg*^*tm1Wjl*^) mice were purchased from Taconic Biosciences (Germantown, NY). *Ptpn11*^fl/fl24^, *Ncr1cre*^Tg25^ and the above-mentioned mice were bred in pathogen-free facilities at Brown University. For MCMV experiments, the ROSA-DTA mice were crossed to *Ncr1cre*^Ki^ mice to generate *Ncr1cre*^Ki^ ROSA-DTA recipient mice, which are deficient in NK cells. Sex- and age-matched 6–12-week-old mice were used for the experiments. The studies performed at Brown University were carried out in strict accordance with the recommendations in the Guide for the Care and Use of Laboratory Animals, as defined by the National Institutes of Health (PHS Assurance #A3284-01). Animal protocols were reviewed and approved by the Institutional Animal Care and Use Committee (IACUC) of Brown University. All animals were housed in a centralized and AAALAC-accredited research animal facility that is fully staffed with trained husbandry, technical, and veterinary personnel.

### Mixed bone marrow chimeras

Donor BM cells were obtained from sex-matched control mice (CD45.1/2) and *Ncr1*^Ki^ *Ptpn11*^fl/fl^ mice (CD45.2) by flushing femurs and tibias and mixed in a 1:1 ratio. A total of 5 × 10^6^ BM cells were injected intravenously into recipient mice (CD45.1), which had been previously lethally irradiated with 900 rad. The recipients were allowed to reconstitute for at least 6 weeks. Frequency of CD45.2 and CD45.1/2 NK cells was determined by flow cytometry.

### Murine lymphocytes isolation

For what concerns *Ncr1*^Ki^*Ptpn11*^fl/fl^ data: splenocytes were obtained by mechanical disruption of the spleen and bone marrow cells were obtained by flushing femurs. Red blood cells were lysed.

For what concerns *Ncr1*^Tg^*Ptpn11*^fl/fl^ data: mice were sacrificed with isoflurane. Cardiac puncture was performed prior to organ removal. Spleens were processed with a GentleMACS Dissociator, filtered through nylon mesh, and layered onto a Lympholyte-M gradient (Cedarlane Laboratories Ltd., Canada). Lymphocytes were harvested from the gradient interface. Livers were perfused before removal, processed in PBS with 1% serum with the GentleMACS, and filtered through nylon mesh. Samples were washed three times and suspended in 40% Percoll and layered on 70% Percoll. Lymphocytes were harvested from the gradient interface and washed once.

### NK cell preparation and culture

For in vitro NK cell experiments, congenically marked splenic NK cells from either control or *Ncr1*^Ki^*Ptpn11*^fl/fl^ mice were isolated using the EasySep mouse NK cell isolation kit (Stemcell Technologies Cat. No. 19855) according to the manufacturer’s recommendations. NK cell enrichment was confirmed by flow cytometry. NK cells were then grown in RPMI 1640 (Life technologies/Cat number 61870010) supplemented with 10% FCS, 100 U/ml penicillin, 100 µg/ml streptomycin, 1 mM sodium pyruvate, 50 µM 2-mercaptoethanol (all from Life technologies), and 10 mM HEPES buffer (Bioconcept) and incubated at 37 °C in 5% CO_2_ with recombinant mouse IL-15 PeproTech/Cat number 210-15) or IL-2 (PeproTech/Cat number 212-12) for 4 or 5 days in the presence or not of Bcl-2 inhibitor (ABT-199 from BioVision), Mcl-1 inhibitor (S63845 from Selleckchem), Shp-2 inhibitor (SHP099 from Selleckchem), the MEK (MAP kinase kinase) inhibitor (PD 98059 from Adipogen), or the mTORC1 inhibitor (Torin2 from Selleckchem) as indicated.

### Cell labeling and in vitro proliferation analysis

Labeling of cells with 5 µM cell trace violet (CTV from Life Technologies) was performed in PBS 1% FCS at 37 °C for 20 min. In order to analyze proliferation, cell divisions were traced by dye dilution at the flow cytometer.

### In vitro killing assay

RMA, RMA-S, and RMA-H60^26^ cell lines were maintained in RPMI 1640 (Life technologies/Cat number 61870010) supplemented with 10% fetal calf serum (FCS, from PAA), 100 U/ml penicillin, 100 µg/ml streptomycin, 50 µM 2-mercaptoethanol (all from Life Technologies) at 37 °C with 5% CO_2_.

For NK cells: control and *Ncr1*^Ki^ *Ptpn11*^fl/fl^ recipient mice were pre-treated with 150 µg of polyinosinic:polycytidylic acid (polyI:C) (InvivoGen) by intraperitoneal injection 1 day before the experiment. Splenocytes were collected after mechanical disruption of the spleen and enriched for NK cells (Stemcell Technologies, Cat. No. 19855). In total, 1 × 10^4^ CTV-labeled target cells (RMA, RMA-S, or RMA-H60) were then co-cultured with NK cells in NK cell medium in a 96-well plate for 4 h in the incubator at 37 °C at the following ratios 10:1, 3:1, or 1:1 NK cells per target. Analysis of the cells was then performed by flow cytometry. RMAs rejection is shown as percentage of killing.

### NK cell degranulation assay

To generate the RMA-m157 transfectants, the retroviral vector pMX encoding MCMV157 ORF was kindly provided by Dr. Heusel (Washington University). RMA target cells were retrovirally transduced and M157 cell surface expression monitored using the anti-M157 mAb Clone 6H1.2.1 (Kindly provided by Dr. Yokoyama Washington University). High expressors for M157 were cell sorted.

For the stimulation with RMA-m157, splenocytes (1 × 10^6^/well) from *Ncr1*^Tg^ *Ptpn11*^fl/fl^ mice or heterozygote littermate controls were incubated for 6 h with 1 × 10^5^ RMA cells or RMA-m157 cells in the presence of Golgi-STOP solution and anti-CD107α antibody. Following incubation, cells were stained with anti-CD3, NK1.1, anti-Ly49H, and NKp46 antibody. Percentage of YFP^+^Ly49H^+^CD107α^+^ NK cells in each group was determined by flow cytometry.

### Seahorse analysis

NK cells were enriched from control and *Ncr1*^Ki^ *Ptpn11*^fl/fl^ mice and stimulated overnight with recombinant mouse IL-15 (50 ng/mL PeproTech) at 37 °C. The following day, 2.5 × 10^5^ enriched NK cells were seeded in a Seahorse Bioscience culture plate in DMEM (Sigma) with 10 mM glucose (Sigma), 2 mM glutamine (Life Technology) in a non-CO_2_ incubator for at least 30 min. Basal OCR and ECAR were then measured by an XF96 Seahorse Extracellular Flux Analyzer following the manufacturer’s instruction. Basal OCR and ECAR were calculated as the mean of the first three time points (basal) for each replicate in *Ncr1*Ki *Ptpn11*^wt/wt^ and *Ncr1*Ki *Ptpn11*^fl/fl^ mice. For the calculation of the SRC, the mean of basal OCR was subtracted to the mean of the three time points at maximal OCR for each replicate in Ncr1Ki Ptpn11^wt/wt^ and Ncr1Ki Ptpn11^fl/fl^ mice.

### NK cell activation in vitro and in vivo

For cytokine stimulation in vitro, splenocytes or enriched NK cells from control and *Ncr1*^Ki^ *Ptpn11*^fl/fl^ mice were left unstimulated or stimulated with recombinant mouse IL-15 (50 ng/mL PeproTech) for 30 min or 1 h at 37 °C and analyzed for intracellular protein phosphorylation by flow cytometry or immunoblot analysis. In some experiments as indicated, NK cells were treated for 30 min with the Shp-2 inhibitor (SHP099 from Selleckchem), the MEK (MAP kinase kinase) inhibitor (PD 98059 from Adipogen), or the mTORC1 inhibitor (Torin2 from Selleckchem) prior to IL-15 stimulation. NK cells were then analyzed by flow cytometry or immunoblot analysis, as described in the specific sections.

For PMA/ionomycin stimulation in vitro, splenocytes from control and *Ncr1*^Ki^ *Ptpn11*^fl/fl^ mice were left unstimulated or stimulated either 15 min with 12.5 nM PMA plus 0.125 µg/ml ionomycin or 2 h with 50 nM PMA plus 0.5 µg/ml ionomycin followed by 2 h with Brefeldin A (10 µg/mL Enzo Life Science) for the detection by flow cytometry of intracellular protein phosphorylation and cytokine production, respectively. In cytokine production experiments, anti-CD107α (LAMP-1) antibody was added at the beginning of incubation to detect NK cell degranulation.

For IL-15 stimulation in vivo, recombinant mouse IL-15Rα-Fc chimeric molecule was purchased from R&D Systems and complexed with recombinant murine IL-15 (PeproTech) for 30 min at 37 °C. Control and *Ncr1*^Ki^ *Ptpn11*^fl/fl^ mice, received 2.5 µg of IL-15 precomplexed with 15 µg of IL-15Rα-Fc in 200 µL of PBS by intraperitoneal injection. Splenic NK cells were analyzed 1 day post-treatment by flow cytometry.

For NK cell activation with purified Ly49H antibody, 96-well flat-bottomed tissue culture plates were coated with purified mouse anti-Ly49H antibody (Biolegend, Clone 3D10, Cat number 144702) or with IgG1 isotope control antibody (Biolegend, Clone MG1-45, Cat number 401404) at 10 µg/ml solution in PBS (without serum) and incubated overnight at 4 °C protected from light. The day after, coated-plates were washed two times with complete medium. In total, 3 × 10^5^ purified NK cells were plated and stimulated for 5 h in the presence or not of 7 ng/ml of IL-15. After 2 h of stimulation, 10 µg/mL of Brefeldin A (Enzo Life Science) was added for the detection by flow cytometry of IFN-γ production.

### Flow cytometry

For flow cytometry analysis, cells were pre-incubated with α-mCD16/32 (2.4G2, 1:100) to block Fc receptors and then surface stained for 20 min at 4 °C using the antibodies listed in Supplementary Table 1. Streptavidin conjugated to different fluorophores were purchased from eBioscience. In some experiments, dead cells were excluded using 10 µg/ml propidium iodide. Events were collected on a FACS Canto, Fortessa, or Aria III (BD) and the data were analyzed using FlowJo software (Tree Star Inc.).

### Intracellular flow cytometry stainings

For detection of phosphorylated signaling proteins, cells were surface stained on ice, fixed with 2% paraformaldehyde at 4 °C for 15 min, and permeabilized with 90% methanol for 30 min at 4 °C. Intracellular phospho-stainings were performed 1 h at room temperature in the dark with antibodies against phospho-STAT5 (Tyr694; D47E7 XP® Rabbit mAb #4322; Cell Signaling, 1:150), phospho-S6 ribosomal protein (Ser235/236; D57.2.2E; XP® Rabbit mAb #4858; Cell Signaling, 1:200), phospho-AKT 308 (Thr308; D25E6; XP® Rabbit mAb #13038; Cell Signaling, 1:100), and phospho-ERK1/2 (Thr202/Tyr204; D13.14.4E; XP® Rabbit mAb #4370; Cell Signaling, 1:150). A secondary antibody coupled to an APC-fluorochrome (Donkey F(ab′)2, anti-rabbit, IgG, multi species, Southern Biotech, 1:250) was used in addition to undirectly conjugated antibodies.

For intracellular staining of Bcl-2 (10C4, eBioscience, 1:100) or Ki67 (SolA15, eBioscience, 1:100), NK cells were first surface stained, fixed and permeabilized using the FoxP3 transcription factor staining buffer set of eBioscience (00-5523-00) according to the manufacturer’s recommendations.

For intracellular cytokine staining, cells were first surface stained, fixed with Cytofix/Cytoperm (BD Biosciences) and permeabilized in 1X PermWash (BD Biosciences) or fixed and permeabilized using a standard PFA/saponin protocol. To conserve eYFP expression during intranuclear staining, samples were prefixed with fresh 4% PFA (Electron Microscopy Science) for 15 min, fixed using Fixation/Permeabilization Solution 2 (Miltenyi Biotech) for 40 min, incubated with 1% Triton X-100 for 15 min, and then stained for 30 min in PBS.

### Immunoblotting

To follow the deletion of Shp-2 in NK cells, splenocytes from *Ncr1*^Tg^ *Ptpn11*^fl/fl^ and control animals were sorted using a FACSAria to >95% efficiency. Populations were collected and lysed in Lysis buffer containing Complete Mini Protease Inhibitor Tablets (Roche Diagnostics). Cellular lysates were added to SDS/Sample Buffer, boiled, and run on a Ready Gel 4–20% Tris-HCl (Bio-Rad, Hercules, CA) with 1× SDS-PAGE Running Buffer. Mouse anti-Shp-2 (M163, Abcam, 1:500) and anti-b-actin antibodies (AC-15, Ambion, 1:1000) were used for immunoblotting. Membranes were developed with West Pico Chemiluminescent Substrate (Pierce) and photographed using a ChemiDoc XRS+ System (Bio-Rad).

Following IL-15 stimulation or under resting conditions to assess the deletion of Shp-2, purified NK cells from *Ncr1*^Ki^*Ptpn11*^fl/fl^ and control mice were collected and resuspended directly in sample buffer containing 62.5 mM Tris-HCL pH 7.4, 2.5% SDS, 0.1 M DTT, 0.002% bromophenol blue, 10% glycerol, 5% 2-mercaptoethanol for protein lysis and extraction. Protein concentration was determined by Coomassie blue staining and equal amounts of proteins were loaded. Rabbit monoclonal antibodies against phospho-STAT5 (Tyr694; D47E7 #4322; Cell Signaling, 1:1000), phospho-S6 (Ser235/236; D57.2.2E #4858; Cell Signaling, 1:2000), phospho-ERK1/2 (Thr202/Tyr204; D13.14.4E #4370; Cell Signaling, 1:2000), Shp-2 (D50F2 #3397; Cell Signaling, 1:1000), STAT5 (D2O6Y #94205; Cell signaling, 1:1000), and mouse monoclonal antibody against S6 (54D2 #2317; Cell Signaling, 1:1000) were used for immunoblotting. The anti-β-actin (from Abcam, 1:2000) polyclonal antibody was used as control. Membranes were revealed using the fusion SOLO S imaging system from Vilber Lourmat.

### MCMV infection

Splenic YFP^+^ NK cells from *Ncr1*^Tg^ *Ptpn11*^fl/fl^ (CD45.2^+^) mice were sorted and injected at a 1:1 ratio with sorted splenic NK cells from B6.SJL (CD45.1^+^) congenic mice into MCMV infected NK cell-deficient mice (*Ncr1cre*^Tg^ROSA-DTA). Stocks of MCMV salivary gland clone RVG-102 (a gift of Dr. Hamilton, Duke University) recombinant for GFP under the promoter of the immediate early gene-1 (ie-1) were generated by homogenizing salivary glands harvested from CD-1 IGS mice (Charles Rivers, stock #022) infected with 5 × 10^4^ PFU of MCMV. Infections were initiated on day 0 with 5 × 10^4^ plaque-forming units (PFU) of MCMV in PBS delivered i.p.

### Adoptive transfer of NK cells and homeostatic proliferation

Splenic YFP^+^ NK cells from *Ncr1*^Tg^ *Ptpn11*^fl/fl^ (CD45.2^+^) mice were sorted and injected at a 1:1 ratio with sorted splenic NK cells from B6.SJL (CD45.1^+^) congenic mice into recipient *Rag2*^*−/*−^*Il2Rg*^−*/*−^ mice. Prior to the injection, NK cells were stained for 10 min at 37 ˚C in the dark with 10 µM Cell Proliferation Dye eFuor450 (eBioscience) in PBS. Recipient mice were allowed to reconstitute for 4 days and sacrificed for analysis.

### Quantitative RT-PCR analysis

BM and splenic NK cells were FACS-sorted as NK1.1^+^NKp46^+^CD3^−^CD19^−^ cells, DP and CD11b SP splenic NK cells were sorted as NK1.1^+^NKp46^+^CD11b^+^CD27^+^CD3^−^CD19^−^ and NK1.1^+^NKp46^+^CD11b^+^CD27^−^CD3^−^CD19^−^ cells, respectively. Sorts were performed using FACSAria, BD Biosciences.

Total RNA of the cells was then extracted using the TRIzol® reagent (Ambion, Life Technologies) according to manufacturer’s instructions. Cellular RNA was extracted using RNeasy kits (Qiagen). Contaminating genomic DNA was removed using RNase-free DNase (Qiagen), and cDNA was synthesized using M-MLV RT, RNase H(–) point mutant (Promega, USA) and Random Primers (Invitrogen). Negative control reactions were performed as above, with the omission of the enzyme or the cDNA. cDNA was quantified using the LightCycler 480 SYBR Green I Master (Roche Diagnostics, Germany) on a LightCycler 480 machine (Roche Diagnostics, Switzerland). Standard cycling was used (45 cycles of 95, 60 and 72 °C-steps of 10 s each). Negative control reactions were cycled alongside test samples to ensure the absence of contaminating genomic DNA. Expression was determined relative to the abundance of the housekeeping gene RNA polymerase II Subunit A (*Polr2a*). Data were analyzed and transcript abundance (*Gene*/*Polr2a*) and SD calculated using the LightCycler 480 software release 1.5.0 SP3. The primers used can be found in Supplementary Table 2.

### Statistical analysis

Statistical analyses were performed using Prism software (GraphPad version 7.0). The Student’s *t*-test (unless otherwise indicated, unpaired, two-tailed) was used to compare the significance of differences between control and Shp-2-deficient conditions, or the indicated experimental groups. Differences considered significant when *p* < 0.05 (*), very significant when *p* < 0.01 (**), highly significant when *p* < 0.001 (***), and extremely significant when *p* < 0.0001 (****).

### Reporting summary

Further information on experimental design is available in the Nature Research Reporting Summary linked to this article.

## Supplementary information

Supplementary Information

Reporting Summary

Source Data

## Data Availability

The data that support the findings of this study are available from the corresponding author upon request.
