# Supplementary material for: Shp-2 is critical for ERK and metabolic engagement downstream of IL-15 receptor in NK cells
Source: Nat Commun. 2019 Mar 29;10:1444. doi: 10.1038/s41467-019-09431-3 (PMC6441079; doi:10.1038/s41467-019-09431-3)
Supplement: Supplementary file 1 — Supplementary Information [file 41467_2019_9431_MOESM1_ESM.pdf]

Shp-2 is critical for ERK and metabolic engagement downstream of IL-15 receptor in NK cells

Niogret et al.

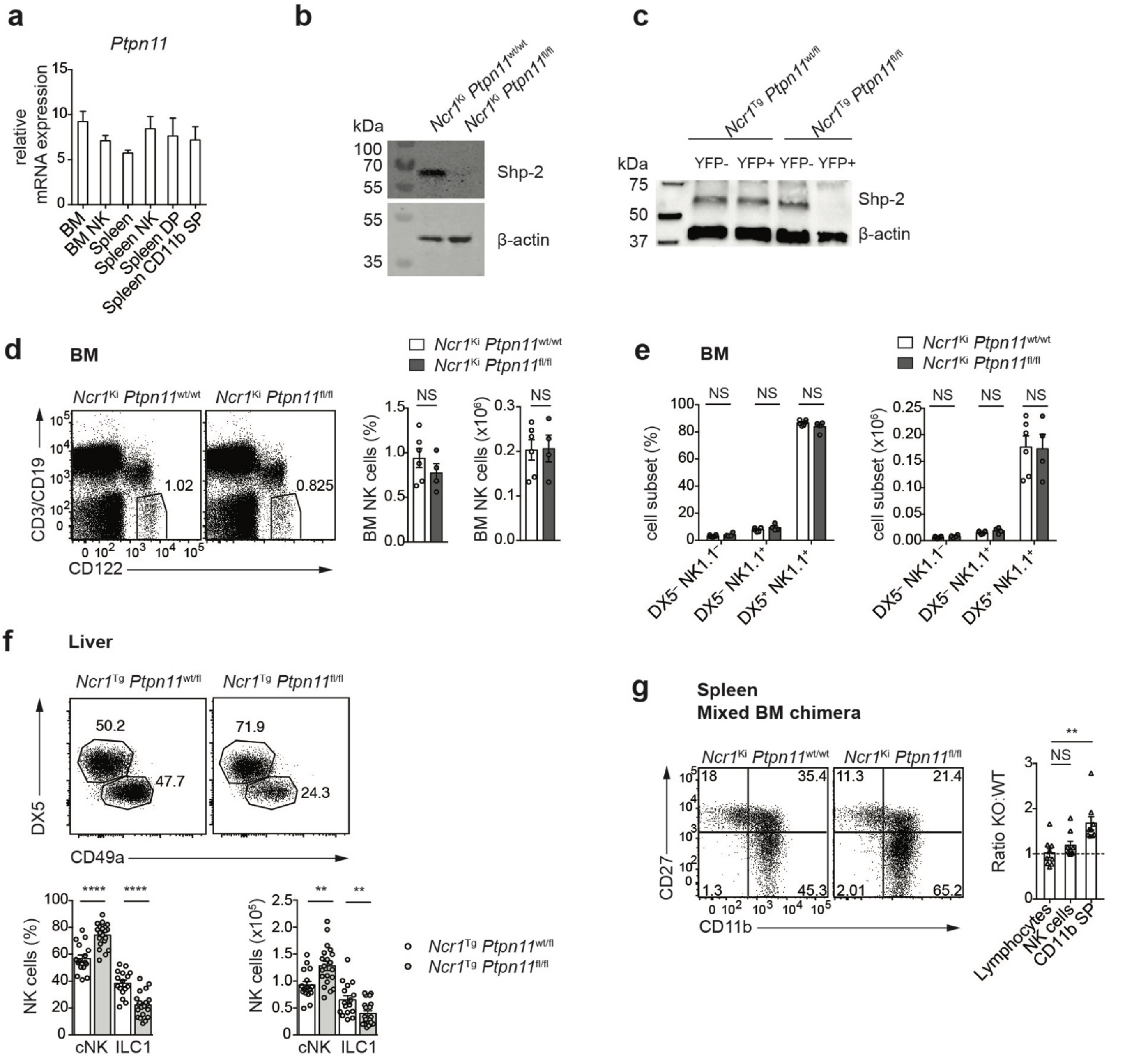

Supplementary Figure 1. Shp-2 is expressed throughout NK cell maturation and can be genetically deleted.

a) qRT-PCR analysis (normalized to *Polr2a*) is shown for *Ptpn11* mRNA in bone marrow (BM) total cells, sorted BM NK cells (NK1.1$^+$NKp46$^+$CD3/19$^-$), total splenocytes, or sorted splenic total NK cells (NK1.1$^+$NKp46$^+$CD3/19$^-$), double-positive (DP) (CD27$^+$CD11b$^+$), and CD11b single-positive (SP) (CD27$^-$CD11b$^+$) subsets from C57BL/6 mice. Results depict mean ± SD (n=3 technical replicates) and are representative of at least two experiments. b and c) Shp-2 protein expression was determined by western blot analysis in NK cells isolated from *Ncr1*^{Ki} *Ptpn11*$^{wt/wt}$ or *Ncr1*^{Ki} *Ptpn11*$^{fl/fl}$ mice (b) or in YFP$^+$NK1.1$^+$CD3$^-$ and YFP$^-$ cells isolated from *Ncr1*^{Tg} *Ptpn11*$^{wt/fl}$ or *Ncr1*^{Tg} *Ptpn11*$^{fl/fl}$ mice (c). Cell lysate was blotted with α-Shp-2 and β-actin antibodies. d and e) Analysis of NK cells from *Ncr1*^{Ki} *Ptpn11*$^{fl/fl}$ (dark grey) and control mice (white). d) A representative flow cytometry image, percentage, and number of NK cells (gated as CD122$^+$CD3/19$^-$) in the BM are shown. e) Development of NK cells (gated on CD122$^+$ CD3/19$^-$) is depicted as percentages and numbers of NK1.1$^-$DX5$^-$, NK1.1$^+$DX5$^-$, and NK1.1$^+$DX5$^+$ in the BM. f) Representative

staining, frequency, and number of conventional NK cells (cNK; DX5$^+$ CD49a–) and innate lymphoid cells 1 (ILC1s; DX5– CD49a$^+$) in the liver of control (white) and *Ncr1*^{Tg} *Ptpn11*$^{fl/fl}$ (light grey) mice, gated as CD45$^+$NK1.1$^+$CD3–YFP$^+$NKp46$^+$. g) Graph depicts the ratio of *Ncr1*^{Ki} *Ptpn11*$^{fl/fl}$ over *Ncr1*^{Ki} *Ptpn11*$^{wt/wt}$ for lymphocytes (normalized to transferred mix), total and CD11b SP NK cells (gated as CD122$^+$NK1.1$^+$CD3/19$^-$) and a representative cytometric plot of Shp-2-deficient and control NK cells stained with CD27 and CD11b in the spleen of mixed BM chimeras. Results represent the mean ± SEM of n=4 (*Ncr1*^{Ki} *Ptpn11*$^{fl/fl}$) and n=6 (*Ncr1*^{Ki} *Ptpn11*$^{wt/wt}$) (d, e), n=16 (*Ncr1*^{Tg} *Ptpn11*$^{wt/fl}$) and n=20 (*Ncr1*^{Tg} *Ptpn11*$^{fl/fl}$) (f), and n=9 (g) mice per genotype and are representative of at least two independent experiments (d-g). Statistical comparison is shown; **p ≤ 0.01, ****p ≤ 0.0001, NS, non-significant; Student's t-test. Source data are provided as a Source Data file.

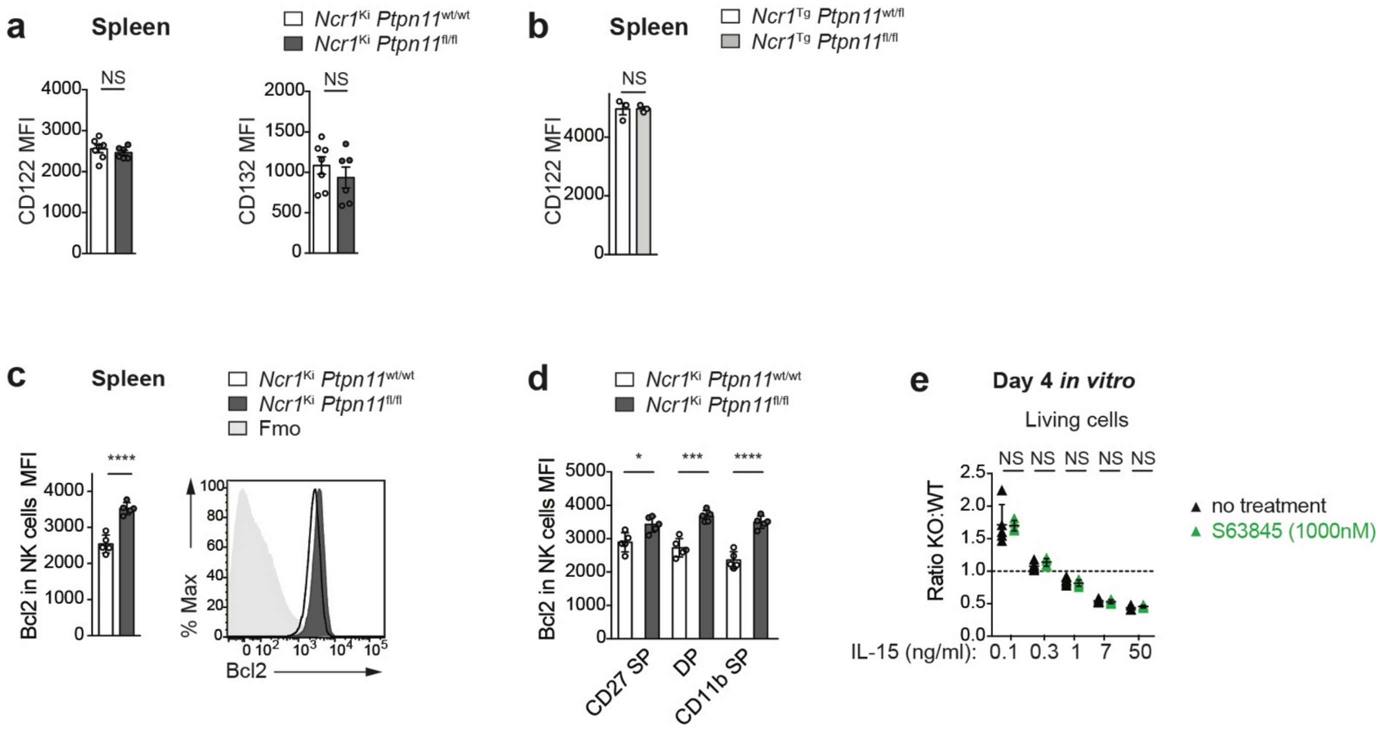

Supplementary Figure 2. Shp-2-deficient NK cells express the IL-15R and modestly increased Bcl-2

a) Geometric MFI of CD122 and CD132 are illustrated for splenic NK cells (gated as NK1.1^{+} and CD3/19^{-}) from *Ncr1*^{Ki} *Ptpn11*$^{wt/wt}$ (white) and *Ncr1*^{Ki} *Ptpn11*$^{fl/fl}$ (dark grey) mice. b) Graph depicts the geometric MFI of CD122 in splenic NK cells from *Ncr1*^{Tg} *Ptpn11*$^{fl/fl}$ (light grey) and control (white) mice. c and d) Geometric mean fluorescence intensity (MFI) of Bcl2 in total (c) and CD27 SP, DP, and CD11b SP (d) splenic NK cells (gated as NK1.1^{+} and CD3/19^{-}) from *Ncr1*^{Ki} *Ptpn11*$^{wt/wt}$ and *Ncr1*^{Ki} *Ptpn11*$^{fl/fl}$ mice. e) Enriched Shp-2-deficient and congenically marked control NK cells were co-cultured for four days in the presence of the indicated amounts of IL-15 and 0 (black triangles) or 1µM S63845 (Mcl-1 inhibitor; green triangles). The graph depicts the ratio of living Shp-2-deficient over control NK cells. Results represent the mean ± SEM of n=6 (*Ncr1*^{Ki} *Ptpn11*$^{fl/fl}$) and n=7 (*Ncr1*^{Ki} *Ptpn11*$^{wt/wt}$) mice (a), n=3 mice (b), and n=5 mice (c,d) per genotype, or results represent mean ± SD of n=4-5 replicates (e) and are representative of at least two independent experiments (a-e). Statistical comparisons are shown; *p ≤ 0.05, ***p ≤ 0.001, ****p ≤ 0.0001, NS, non-significant; Student's t-test. Source data are provided as a Source Data file.

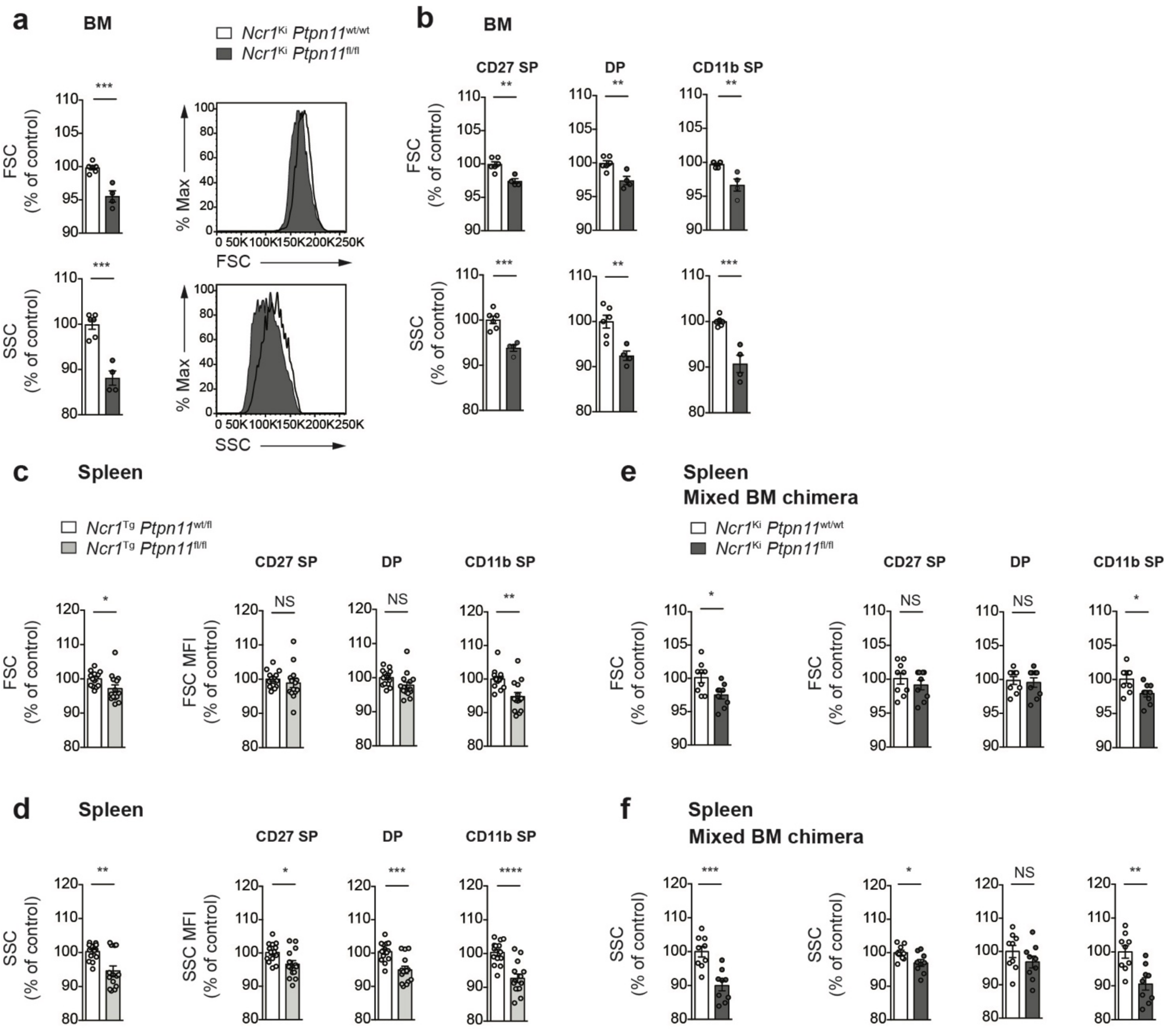

Supplementary Figure 3. Shp-2 maintains size and granularity in an NK cell-intrinsic manner

a, b) Representative cytometric profile and quantification of FSC and SSC for total (a) and CD27 SP, DP, and CD11b SP NK cell populations (b) in the BM (gated as $CD122^{+}NK1.1^{+}NKp46^{+}CD3/19^{-}$) from *Ncr1*^{Ki} *Ptpn11*$^{\text{wt/wt}}$ (white) and *Ncr1*^{Ki} *Ptpn11*$^{\text{fl/fl}}$ (dark grey). For quantification, the average of *Ncr1*^{Ki} *Ptpn11*$^{\text{wt/wt}}$ was set as 100%. c, d) Analysis of NK cells in the spleen (gated as $NK1.1^{+}CD3^{-}YFP^{+}NKp46^{+}$) of *Ncr1*^{Tg} *Ptpn11*$^{\text{fl/fl}}$ (light grey) and control (white) mice. Quantification of forward scatter (FSC) (c) and side scatter (SSC) (d) for total and CD27 SP, DP, and CD11b SP NK cells (average of *Ncr1*^{Tg} *Ptpn11*$^{\text{wt/fl}}$ was set as 100%). e, f) Analysis of FSC (e) and SSC (f) for total and CD27 SP, DP, and CD11b SP NK cells in the spleen of *Ncr1*^{Ki} *Ptpn11*$^{\text{wt/wt}}$: *Ncr1*^{Ki} *Ptpn11*$^{\text{fl/fl}}$ mixed BM chimeras (average of *Ncr1*^{Ki} *Ptpn11*$^{\text{wt/wt}}$ was set as 100%). Results represent the mean ± SEM of n=4 (*Ncr1*^{Ki} *Ptpn11*$^{\text{fl/fl}}$) and n=6 (*Ncr1*^{Ki} *Ptpn11*$^{\text{wt/wt}}$) mice (a,b), n=14 mice per genotype (c, d), and n=9 mice (e, f) and are a pool of four experiments (c, d) and representative of at least two (a, b, e, and f) independent experiments. Statistical comparison are shown; $*p \leq 0.05$, $**p \leq 0.01$, $***p \leq 0.001$, $****p \leq 0.0001$, NS, non-significant; Student's t-test. Source data are provided as a Source Data file.

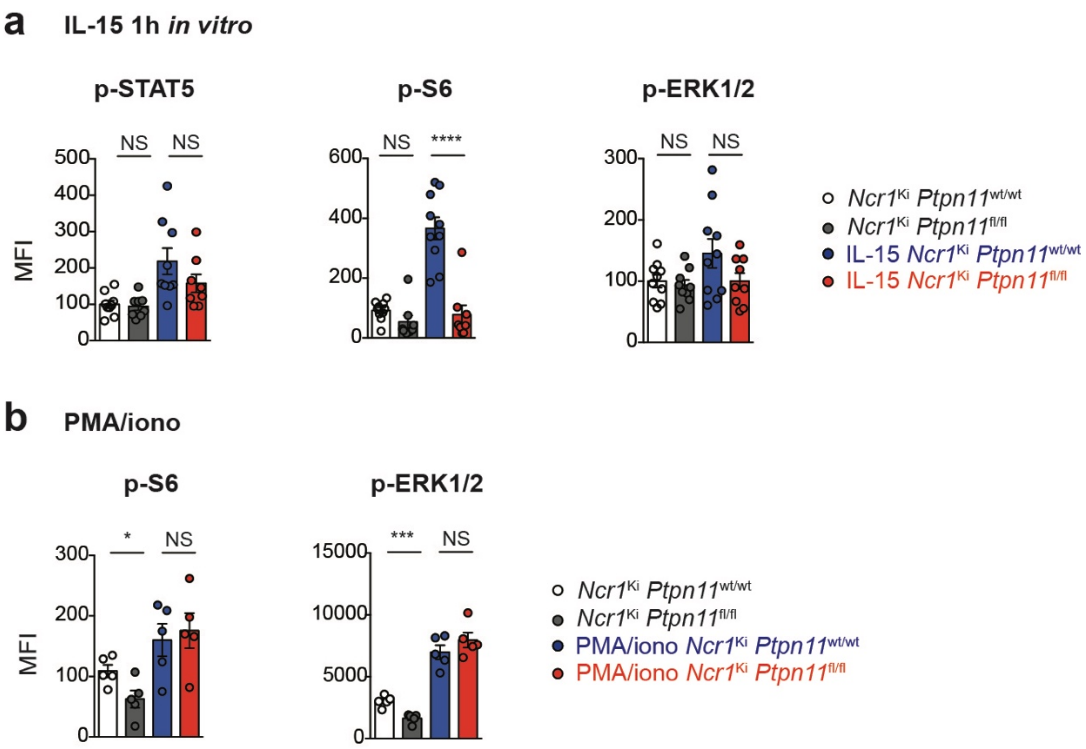

Supplementary Figure 4. Shp-2-deficient NK cells normally phosphorylate ERK and S6 in response to PMA/ionomycin

a) Splenocytes from *Ncr1*^{Ki} Ptpn11$^{wt/wt}$ and *Ncr1*^{Ki} Ptpn11$^{fl/fl}$ mice were cultured *in vitro* for one hour in the presence of 50 ng/ml IL-15 (blue and red, respectively) or left untreated (white and dark grey, respectively). Phosphorylation of STAT5, S6, and ERK were measured by flow cytometry (as geometric MFI) in NK cells (gated as NK1.1^{+} and CD3/19^{-}). For quantification, the average of the untreated NK cells from *Ncr1*^{Ki} *Ptpn11*$^{wt/wt}$ mice was set as 100%. b) Phosphorylation of ERK and S6 in splenic NK cells (gated as NK1.1^{+} and CD3/19^{-}) from *Ncr1*^{Ki} *Ptpn11*$^{wt/wt}$ and *Ncr1*^{Ki} *Ptpn11*$^{fl/fl}$ mice following 15 minutes stimulation with PMA/ionomycin. Results represent the mean ± SEM of n=8-9 (*Ncr1*^{Ki} *Ptpn11*$^{fl/fl}$) and n=9-10 (*Ncr1*^{Ki} *Ptpn11*$^{wt/wt}$) (a), of n=5 (b) mice per genotype and are a pool of 3 independent experiments (a) and are representative of at least two independent experiments (b). Statistical comparisons are shown; *p ≤ 0.05, ***p ≤ 0.001, ****p ≤ 0.0001, NS, non-significant; Student's t-test. Source data are provided as a Source Data file.

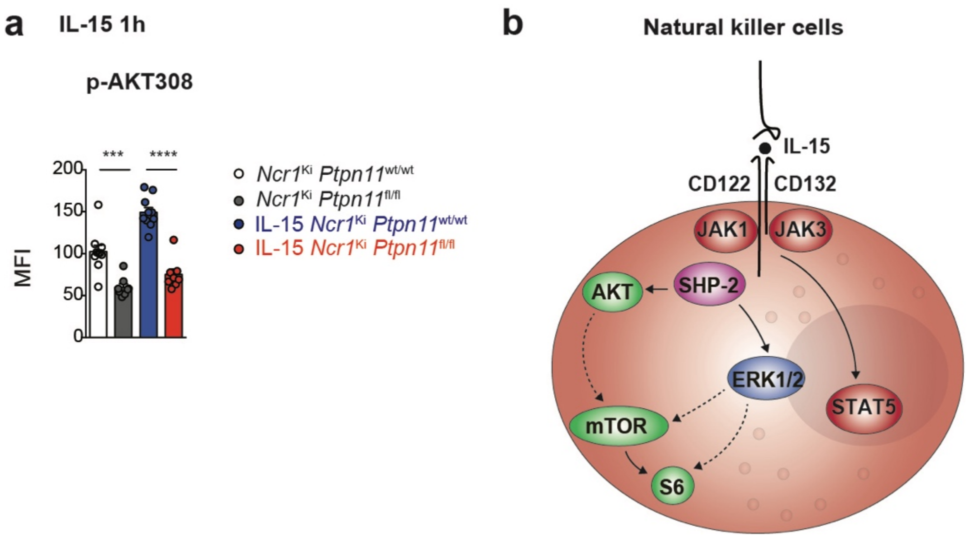

Supplementary Figure 5. Shp-2 regulates IL-15-dependent Akt phosphorylation

a) Splenocytes from *Ncr1*^{Ki} Ptpn11$^{wt/wt}$ and *Ncr1*^{Ki} Ptpn11$^{fl/fl}$ mice were cultured *in vitro* for one hour in the presence of 50 ng/ml IL-15 (blue and red, respectively) or left untreated (white and dark grey, respectively). Phosphorylation of Akt (position 308) was measured by flow cytometry (as geometric MFI) in NK cells (gated as NK1.1^{+} and CD3/19^{-}). For quantification, the average of the untreated NK cells from *Ncr1*^{Ki} *Ptpn11*$^{wt/wt}$ mice was set as 100%. Results represent the mean ± SEM of n=8 (*Ncr1*^{Ki} *Ptpn11*$^{fl/fl}$) and n=10 (*Ncr1*^{Ki} *Ptpn11*$^{wt/wt}$) mice per genotype and are a pool of 3 independent experiments (a). Statistical comparisons are shown; ***$p \leq 0.001$, ****$p \leq 0.0001$; Student's t-test. Source data are provided as a Source Data file. b) Schematic representation of the role of Shp-2 downstream of IL-15R in NK cells.

a Ly49H stimulation 5 hours

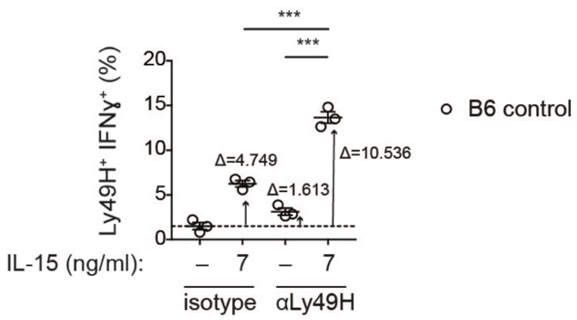

Supplementary Figure 6. Ly49H stimulation potentiates IL-15-induced IFNγ production by NK cells

a) Plate-bound anti-Ly49H (mAb 3D10) or isotype control mAb were used to stimulate enriched control splenic NK cells from C57/BL6 wild type mice in the presence or not of 7ng/mL of IL-15 for 5 hours. Graph depicts the percentage of IFNγ$^+$ among Ly49H$^+$ NK cells (gated on Ly49H$^+$ NK1.1$^+$ and CD3/CD19$^-$) after 5 hours of stimulation. Results represent mean ± SEM of n=3 independent experiments (the average of each experiment is shown). The difference to basal IFNγ$^+$ cell percentage in each condition is indicated. Statistical comparisons are shown; ***$p \leq 0.001$; Student's t-test. Source data are provided as a Source Data file.

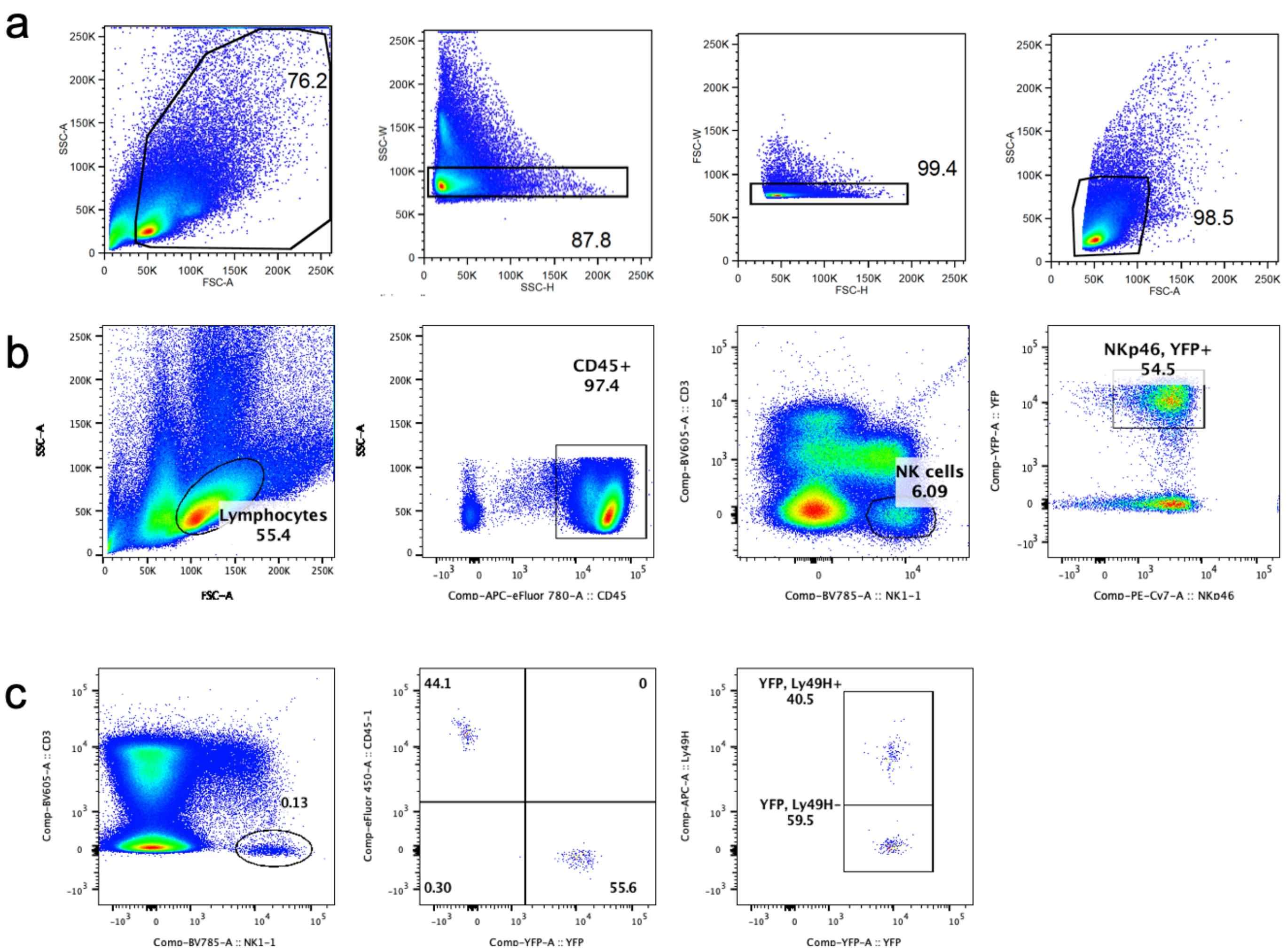

Supplementary Figure 7. Gating strategies for flow cytometry analysis

a-c) The gating strategies illustrated above were performed for gating on NK cells. a) Unless otherwise indicated, this is a representative example of gating strategy used in combination with those illustrated in Figure 1a-e for measuring extracellular and intracellular stainings on NK cells/NK cell subsets. b) Gating strategy used for Fig.1f and Supplementary Fig.1f. c) Gating strategy used for Fig. 8; gating on Ly49H was used only for panels c and d.

Supplementary Table 1. Antibodies used for flow cytometry

Antibody target	Clone name	Source	Dilution
CD3	145-2C11	eBioscience, Biolegend	1:100
CD3	17A2	eBioscience, Biolegend	1:200
CD11b	M1/70	eBioscience, Biolegend	1:250
CD19	1D3	eBioscience, Biolegend	1:200
CD19	6D5	eBioscience, Biolegend Miltenyi Biotec,	1:200
CD27	LG.7F9	eBioscience	1:100
CD27	LG3A10	Biolegend	1:100
CD45	30-F11	eBioscience, Biolegend	1:100
CD45.1	A20	eBioscience, Biolegend	1:100
CD45.2	104	eBioscience, Biolegend	1:100
CD49a	HMα1	Biolegend	1:200
DX5/CD49b	DX5	eBioscience, Biolegend	1:100
CD94	18D3	eBioscience, Biolegend	1:100
CD107α	1D4B	eBioscience, Biolegend	1:400
CD122	TM-b1	eBioscience, Biolegend	1:100
CD132	TUGm2	eBioscience	1:100
granzyme A	GzA-3G8.5	eBioscience	1:100
granzyme B	NGZB	eBioscience	1:75
IFN-γ	XMG1.2	eBioscience	1:100
KLRG1	2F1	eBioscience, Biolegend	1:200
Ly49A	A1	eBioscience, Biolegend	1:100
Ly49D	4E5	eBioscience	1:100
Ly49G2	4D11	eBioscience	1:100
Ly49H	3D10	Biolegend	1:100
Ly49I	YLI-90	eBioscience	1:100
NK1.1	PK-136	eBioscience, Biolegend	1:100
NKp46	29A1.4	eBioscience, Biolegend	1:100
TCRβ	H57-597	eBioscience, Biolegend	1:200

Supplementary Table 2. Primer sequences for RT-PCR analysis

Mouse gene	Forward	Reverse
Ptpn11	AGACTTCGTTCTCTCCGTGC	CTGTCAGAGAGTCAAAGCGC
Polr2a	CCGGATGAATTGAAGCGGATGT	CCTGCCGTGGATCCATTAGTCC
