# Supplementary material for: Shp-2 is critical for ERK and metabolic engagement downstream of IL-15 receptor in NK cells
Source: Nat Commun. 2019 Mar 29;10:1444. doi: 10.1038/s41467-019-09431-3 (PMC6441079; doi:10.1038/s41467-019-09431-3)
Supplement: Supplementary file 2 — Reporting Summary [file 41467_2019_9431_MOESM2_ESM.pdf]

nature research

Corresponding author(s): Greta Guarda and Laurent Brossay

Reporting Summary

Nature Research wishes to improve the reproducibility of the work that we publish. This form provides structure for consistency and transparency in reporting. For further information on Nature Research policies, see Authors & Referees and the Editorial Policy Checklist.

Statistical parameters

When statistical analyses are reported, confirm that the following items are present in the relevant location (e.g. figure legend, table legend, main text, or Methods section).

n/a	Confirmed	
☐	☒	The exact sample size (n) for each experimental group/condition, given as a discrete number and unit of measurement
☐	☒	An indication of whether measurements were taken from distinct samples or whether the same sample was measured repeatedly
☐	☒	The statistical test(s) used AND whether they are one- or two-sided<br>*Only common tests should be described solely by name; describe more complex techniques in the Methods section.*
☒	☐	A description of all covariates tested
☒	☐	A description of any assumptions or corrections, such as tests of normality and adjustment for multiple comparisons
☐	☒	A full description of the statistics including central tendency (e.g. means) or other basic estimates (e.g. regression coefficient) AND variation (e.g. standard deviation) or associated estimates of uncertainty (e.g. confidence intervals)
☒	☐	For null hypothesis testing, the test statistic (e.g. F, t, r) with confidence intervals, effect sizes, degrees of freedom and P value noted<br>*Give P values as exact values whenever suitable.*
☒	☐	For Bayesian analysis, information on the choice of priors and Markov chain Monte Carlo settings
☒	☐	For hierarchical and complex designs, identification of the appropriate level for tests and full reporting of outcomes
☒	☐	Estimates of effect sizes (e.g. Cohen's d, Pearson's r), indicating how they were calculated
☐	☒	Clearly defined error bars<br>*State explicitly what error bars represent (e.g. SD, SE, CI)*

Our web collection on statistics for biologists may be useful.

Software and code

Policy information about availability of computer code

Data collection	N/A
Data analysis	N/A

For manuscripts utilizing custom algorithms or software that are central to the research but not yet described in published literature, software must be made available to editors/reviewers upon request. We strongly encourage code deposition in a community repository (e.g. GitHub). See the Nature Research guidelines for submitting code & software for further information.

Data

Policy information about availability of data

All manuscripts must include a data availability statement. This statement should provide the following information, where applicable:

- Accession codes, unique identifiers, or web links for publicly available datasets
- A list of figures that have associated raw data
- A description of any restrictions on data availability

The datasets generated during and/or analysed during the current study are available from the corresponding authors on reasonable request.

Field-specific reporting

Please select the best fit for your research. If you are not sure, read the appropriate sections before making your selection.

☒ Life sciences ☐ Behavioural & social sciences ☐ Ecological, evolutionary & environmental sciences

For a reference copy of the document with all sections, see nature.com/authors/policies/ReportingSummary-flat.pdf

Life sciences study design

All studies must disclose on these points even when the disclosure is negative.

Sample size	Since this study assessed the function of a protein uncharacterized in NK cells, we could not anticipate differences and variability of our experiments, and therefore the sample size was not predetermined. Usually, a first experiment was performed with 3 to 4 mice / group to determine the number of replicates to use in later analyses, as a function of the mean and the standard deviation. Based on the pilot experiment, the sample size of the groups was either maintained in a similar range or increased/pooled.
Data exclusions	No data exclusion were applied except in the following cases:<br>1/ Technical issues: Data were not considered unless all quality controls included in the experiment were met;<br>2/ For quantitative PCR/RT-PCR, outlier values within triplicates were excluded (cf. Current protocols in molecular biology)
Replication	For experiments that required technical troubleshootings, data were not considered until all quality controls were met. Experiments were repeated as reported in the figure legends and were reliably reproduced.
Randomization	Randomisation was not relevant for our purpose. As we aimed at identifying the function of Shp-2 in NK cells, all experimental groups were constituted according to the genotypes of the animals or cells.
Blinding	N/A. Blinding was not relevant for our purpose.

Reporting for specific materials, systems and methods

Materials & experimental systems

n/a	Involved in the study
☒	☐ Unique biological materials
☐	☒ Antibodies
☐	☒ Eukaryotic cell lines
☒	☐ Palaeontology
☐	☒ Animals and other organisms
☒	☐ Human research participants

Methods

n/a	Involved in the study
☒	☐ ChIP-seq
☐	☒ Flow cytometry
☒	☐ MRI-based neuroimaging

Antibodies

Antibodies used	(1) Western blot: α-phospho-STAT5 (Tyr694; D47E7 #4322; Cell signaling), phospho-S6 (Ser235/236; D57.2.2E #4858; Cell signaling), phospho-ERK1/2 (Thr202/Tyr204; D13.14.4E #4370; Cell signaling), Shp-2 (D50F2 #3397; Cell signaling or M163, Abcam), STAT5 (D2O6Y #94205; Cell signaling), S6 (54D2 #2317; Cell signaling), anti-b-actin (from Abcam or AC-15 Ambion)<br>(2) Flow cytometry: α-Bcl-2 (10C4),CD16/32 (2.4G2),CD3e (145-2C11), CD3 (17A2),CD11b (M1/70), CD19 (1D3 or 6D5), CD27 (LG.7F9 or LG3A10), CD45 (30-F11), CD45.1 (A20), CD45.2 (104), CD49a (HMa1), DX5/CD49b (DX5), CD94 (18D3), CD107a (1D4B), CD122 (TM-b1), CD132 (TUGm2), granzyme A (GzA-3G8.5), granzymes B (NGZB), IFN-γ (XMG1.2), Ki67 (SolA15), KLRG1 (2F1), Ly49A (A1), Ly49D (4E5), Ly49G2 (4D11), Ly49H (3D10), Ly49I (YLI-90), NK1.1 (PK-136), NKp46 (29A1.4), TCRβ (H57-597 (all from eBioscience, Biolegend, or Invitrogen), phospho-AKT 308 (Thr308;D25E6 XP® Rabbit mAb #13038), phospho-STAT5 (Tyr694;D47E7 XP® Rabbit mAb #4322), phospho-S6 ribosomal protein (Ser235/236; D57.2.2E; XP® Rabbit mAb #4858) and phospho-ERK1/2 (Thr202 Tyr204; D13.14.4E; XP® Rabbit mAb #4370) from Cell signaling, an APC fluorochrome (Donkey F(ab')2, anti-rabbit, IgG, multi species) from Southern Biotech, and CD19 (65D) from Miltenyi Biotec.
Validation	Commercially available antibodies were used and were previously validated and/or commonly used in publications.

Eukaryotic cell lines

Policy information about cell lines

Cell line source(s)	RMA, RMA-S, and RMA-H60 were obtained from Werner Held , Department of Oncology UNIL CHUV, University of Lausanne, Lausanne, Switzerland<br>RMA target cells were retrovirally transduced to generate RMA-m157 cells. RMA cells were obtained from Dr. Kronenberg laboratory, La Jolla Institute for Immunology 9420 Athena Circle La Jolla, CA 92037Tel: +1 (858) 752-6500.
Authentication	No formal authentification of the cell line have been performed
Mycoplasma contamination	Not checked.
Commonly misidentified lines (See ICLAC register)	No commonly misidentified cell lines were used

Animals and other organisms

Policy information about studies involving animals; ARRIVE guidelines recommended for reporting animal research

Laboratory animals	NK cell-specific knockout mice for Ptpn11 were generated by crossing either the Ptpn11fl/fl mice15 to the Ncr1creKi deleter strain23 or the Ptpn11fl/fl mice24 to the Ncr1creTg mice61, generating Ncr1KiPtpn11fl/fl and Ncr1Tg Ptpn11fl/fl, respectively. Ncr1creTg Ptpn11fl/fl mice were crossed to R26R-EFYP mice to monitor cre activity.<br>For genotyping Ncr1KiPtpn11fl/fl mice, Ptpn11-floxed alleles were detected by PCR amplification using a set of 2 primers that amplify a 300 bp wt band and a 380 bp floxed band (forward primer 5'-ATGACTCCTGAAGCCCATTG-3' and reverse primer 5'-TTCCCATCACCTCAGACTCC - 3'). For genotyping Ncr1Tg Ptpn11fl/fl mice, Ptpn11-floxed alleles were detected by PCR amplification using a set of 2 primers that amplify a 312 bp wt band and a 430 bp floxed band (forward primer 5'-TAGCTGCTTTAACCCTCTGTGT-3'; reverse primer 5'-CATCAGAGCAGGCCATATTCC-3')<br>Ptpn11fl/fl15, Ncr1creKi, B6.SJL, β2m-/-, and C57BL/6 mice, all on a C57BL/6 (H2b) background, were bred under specific pathogen-free conditions at the animal facility of the University of Lausanne. Sex and age-matched 6 to 14 week-old mice were used for the experiments. All animal experimental protocols were approved by the Veterinary office regulations of the State of Vaud, Switzerland, and all methods were performed in accordance with the Swiss guidelines and regulations.<br>C57BL/6, B6.SJL (B6.SJL-Ptprca Pepcb/BoyJ), R26R-EFYP (B6.129X1 Gt(ROSA)26Sortm1(EYFP)Cos/J), and ROSA-DTA (B6.129P2-Gt(ROSA)26Sortm1(DTA)Lky/J) mice were purchased from The Jackson Laboratory (Bar Harbor, ME). Rag2-/-Il-2Rg-/- (B10;B6-Rag2tm1Fwa Il2rgtm1Wjl) mice were purchased from Taconic Biosciences (Germantown, NY). Ptpn11fl/fl24, Ncr1creTg25 and the above-mentioned mice were bred in pathogen-free facilities at Brown University. For MCMV experiments, the ROSA-DTA mice were crossed to Ncr1creKi mice to generate Ncr1creKiROSA-DTA recipient mice, which are deficient in NK cells. Sex and age-matched 6 to 12 week-old mice were used for the experiments.
Wild animals	N/A
Field-collected samples	N/A

Flow Cytometry

Plots

Confirm that:

- [ ] The axis labels state the marker and fluorochrome used (e.g. CD4-FITC).
- [x] The axis scales are clearly visible. Include numbers along axes only for bottom left plot of group (a 'group' is an analysis of identical markers).
- [ ] All plots are contour plots with outliers or pseudocolor plots.
- [x] A numerical value for number of cells or percentage (with statistics) is provided.

Methodology

Sample preparation	For what concerns Ncr1Ki Ptpn11fl/fl data: mice were euthanized, and tibia+femurs and spleen were collected from mice. Cell suspensions from spleen were prepared by mashing the organ on a filter tissue in PBS and treated with red blood cell lysis buffer during 5 minutes at room temperature (RT), washed, resupended in PBS, and counted. For BM lymphocytes, the BM was flushed out from the femur and tibia with PBS, 2% FCS and were passed through a 26 gauge needle. The cell suspension was then treated with red blood cell lysis buffer for 5 minutes, washed, resuspended in PBS, and counted.<br><br>For what concerns Ncr1Tg Ptpn11fl/fl data: mice were sacrificed and spleen and liver were collected from mice. Cardiac puncture was performed prior to organ removal. Spleens were processed with a GentleMACS Dissociator, filtered through nylon mesh, and layered onto a Lympholyte-M gradient (Cedarlane Laboratories Ltd., Canada). Lymphocytes were harvested from the gradient interface. Livers were perfused before removal, processed in PBS with 1% serum with the GentleMACS, and filtered through nylon mesh. Samples were washed 3 times and suspended in 40% Percoll and layered on 70% Percoll. Lymphocytes were harvested from the gradient interface and washed once.

Instrument	For flow cytometry (FC) analyses Beckton Dickinsons (BD) analyzers were used; FACSCanto, FACSFortessa, LSR II, or Aria III (Special Order and Research Product). All sorts were performed in the BD FACSAria II or FACSAria III.
Software	FC data were collected on a BD Biosciences flow cytometer using BD FACSDiva™ software.<br>The analysis of flow cytometry data was performed using FlowJo software version 9.9.6 or 10.4.2.
Cell population abundance	For qPCR analysis of Ptpn11 mRNA expression: BM and splenic NK cells were FACS-sorted as NK1.1+NKp46+CD3-CD19- cells, DP and CD11b SP splenic NK cells were sorted as NK1.1+NKp46+CD11b+CD27+CD3-CD19- and NK1.1+NKp46+CD11b+CD27-CD3-CD19- cells, respectively. Purity of the sorted cells was determined by running a fraction of the sorted sample and was equal or superior to 99 %.<br>For NK cell transfer experiments: Splenic YFP+ NK cells were FACS-sorted as CD3-NK1.1+NKp46+YFP+ cells. Purity of the sorted cells was determined by running a fraction of the sorted sample and was equal or superior to 98 %. Splenic CD45.1 congenic NK cells were FACS-sorted as CD3-NK1.1+NKp46+CD45.1+ cells. Purity of the sorted cells was determined by running a fraction of the sorted sample and was equal or superior to 98 %.
Gating strategy	Flow cytometry data analysis was performed by excluding debris and doublets; then a relatively large gate was placed around the lymphocyte population according to forward scatter and side scatter (Supplementary Figure 7). To investigate and to quantify NK cell populations either NK1.1 or CD122 versus lineage (CD3 and CD19) was used as illustrated in Fig. 1 and Supplementary Fig. 1.

☒ Tick this box to confirm that a figure exemplifying the gating strategy is provided in the Supplementary Information.
